# Adaptation of targeted nanocarriers to changing requirements in antimalarial drug delivery

**DOI:** 10.1016/j.nano.2016.09.010

**Published:** 2017-02

**Authors:** Joana Marques, Juan José Valle-Delgado, Patricia Urbán, Elisabet Baró, Rafel Prohens, Alfredo Mayor, Pau Cisteró, Michael Delves, Robert E. Sinden, Christian Grandfils, José L. de Paz, José A. García-Salcedo, Xavier Fernàndez-Busquets

**Affiliations:** a Nanomalaria Group, Institute for Bioengineering of Catalonia (IBEC), Barcelona, Spain; b Barcelona Institute for Global Health (ISGlobal), Barcelona Center for International Health Research (CRESIB, Hospital Clínic-Universitat de Barcelona), Barcelona, Spain; c Nanoscience and Nanotechnology Institute (IN2UB), University of Barcelona, Barcelona, Spain; d Unitat de Polimorfisme i Calorimetria, Centres Científics i Tecnològics, Universitat de Barcelona, Barcelona, Spain; e Department of Life Sciences, Imperial College, South Kensington, London, UK; f Interfacultary Research Center of Biomaterials (CEIB), University of Liège, Chemistry Institute, Liège (Sart-Tilman), Belgium; g Instituto de Investigaciones Químicas (IIQ) CSIC-US, Centro de Investigaciones Científicas Isla de La Cartuja, Sevilla, Spain; h Unidad de Enfermedades Infecciosas y Microbiología, Instituto de Investigación Biosanitaria ibs. Granada, Hospitales Universitarios de Granada/Universidad de Granada, Granada, Spain

**Keywords:** Glycosaminoglycans, Malaria, Nanomedicine, *Plasmodium*, Targeted drug delivery

## Abstract

The adaptation of existing antimalarial nanocarriers to new *Plasmodium* stages, drugs, targeting molecules, or encapsulating structures is a strategy that can provide new nanotechnology-based, cost-efficient therapies against malaria. We have explored the modification of different liposome prototypes that had been developed in our group for the targeted delivery of antimalarial drugs to *Plasmodium*-infected red blood cells (pRBCs). These new models include: (i) immunoliposome-mediated release of new lipid-based antimalarials; (ii) liposomes targeted to pRBCs with covalently linked heparin to reduce anticoagulation risks; (iii) adaptation of heparin to pRBC targeting of chitosan nanoparticles; (iv) use of heparin for the targeting of *Plasmodium* stages in the mosquito vector; and (v) use of the non-anticoagulant glycosaminoglycan chondroitin 4-sulfate as a heparin surrogate for pRBC targeting. The results presented indicate that the tuning of existing nanovessels to new malaria-related targets is a valid low-cost alternative to the *de novo* development of targeted nanosystems.

## Graphical Abstract

Preexisting antimalarial nanocarriers and targeting molecules (gray boxes) have been modified in their nanocapsule, targeting molecule and drug cargo to adapt them to new therapeutic strategies against malaria parasites

**Figure:**
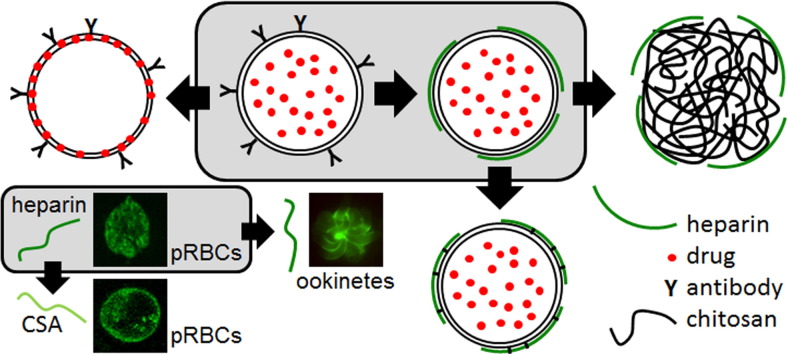

Antimalarial drugs can potentially target a suite of pathogen life stages inside two different hosts: humans and the insect vectors. Infection starts when a parasitized female *Anopheles* mosquito inoculates sporozoites of the malaria parasite, the protist *Plasmodium* spp., into a person while taking a blood meal. Within a few minutes, sporozoites have migrated through the skin and bloodstream to the liver, where they invade hepatocytes. Sporozoites develop into merozoites,^1^ which enter the circulation, invade red blood cells (RBCs),^2^ and replicate asexually to produce daughter cells that invade new RBCs to perpetuate the blood-stage cycle unfolding through ring, trophozoite, and schizont stages. Some parasites eventually differentiate into sexual stages, female and male gametocytes that are ingested by a mosquito from peripheral blood. When an infected bloodmeal reaches the insect's midgut, micro- and macrogametocytes develop into male and female gametes. Following fertilization, the zygote differentiates into an ookinete that moves through the midgut epithelium and forms an oocyst, which releases sporozoites. The malaria transmission cycle is restarted when sporozoites migrate to the salivary glands and are injected into a human with the mosquito's next bite.

With malaria elimination now firmly on the global research agenda, but resistance to the currently available drugs on the rise, there is an urgent need to invest in research and development of new therapeutic strategies.^3^ Encapsulation of drugs in targeted nanovectors is a rapidly growing area with a clear applicability to infectious disease treatment,^4^ and pharmaceutical nanotechnology has been identified as a potentially essential tool in the future fight against malaria.^5^, ^6^ Nanoparticle-based targeted delivery approaches can play an important role for the treatment of malaria because they might allow (i) low overall doses that limit the toxicity of the drug for the patient, (ii) administration of sufficiently high local amounts to minimize the evolution of resistant parasite strains,^7^ (iii) improvement of the efficacy of currently used hydrophilic (low membrane trespassing capacity) and lipophilic antimalarials (poor aqueous solubility), and (iv) use of orphan drugs never assayed as malaria therapy, *e.g.* because of their elevated and wide-spectrum toxicity. In the very nature of nanovectors resides their versatility that enables assembling several elements to obtain chimeric nanovessels tailored to fit the requirements for different administration routes, particular intracellular targets, or combinations of drugs.

One of the limitations of liposomes as carriers for drug delivery to *Plasmodium*-infected RBCs (pRBCs) is that because of the lack of endocytic processes in these cells, a relatively fluid liposome lipid bilayer is required to favor fusion events with the pRBC plasma membrane. As a result, these liposomes are leaky for small drugs encapsulated in their lumen,^8^ and when membrane fusion occurs, only a relatively small fraction of the originally contained drug is delivered into the cell. On the other hand, liposomes made of saturated lipids have less fluid bilayers that retain drugs with high efficacy,^8^ although fusion events with pRBC membranes are greatly diminished, which might also reduce the amount of luminal cargo delivered to the target cell. The so-called combination therapies, where several drugs are simultaneously administered,^9^ significantly improve the antimalarial effect of the individual compounds. Liposomes are particularly adept structures in this regard because they allow the encapsulation of hydrophobic molecules in their lipid bilayer and of water-soluble compounds in their lumen, thus being a potentially interesting platform for combination therapies where lipophilic and hydrophilic drugs are delivered together.

One of the main pRBC-binding molecules are glycosaminoglycans (GAGs), some of whose members include heparin, heparan sulfate (HS), and chondroitin sulfate (CS). Chondroitin 4-sulfate (CSA) has been found to act as a receptor for pRBC binding in the microvasculature and the placenta,^10^ and adhesion of pRBCs to placental CSA has been linked to the severe disease outcome of pregnancy-associated malaria.^11^ pRBC adhesion to the endothelium of postcapillary venules is mediated by the parasite-derived antigen *Plasmodium falciparum* erythrocyte membrane protein 1 (PfEMP1),^12^ whereas CSA has been identified as the main receptor for PfEMP1 attachment to placental cells.^10^, ^13^ Single-molecule force spectroscopy data have revealed a complete specificity of adhesion of heparin to pRBCs *vs.* RBCs, with a binding strength matching that of antibody–antigen interactions.^14^ Heparin had been used in the treatment of severe malaria,^15^ but it was abandoned because of its strong anticoagulant action, with side effects such as intracranial bleeding. It has been shown that heparin electrostatically bound to liposomes acts as an antibody surrogate, having a dual activity as a pRBC targeting molecule but also as an antimalarial drug in itself acting mainly on trophozoite and schizont stages.^16^ Because heparin is significantly less expensive to obtain than specific (monoclonal) antibodies, the resulting heparin-liposomes have a cost about ten times lower than that of equally performing immunoliposomes. A question that remains open is whether the heparin-mediated targeting of liposomes to pRBCs could be extended to other glycosaminoglycans, to different *Plasmodium* stages, and to new nanoparticle types.

Through modification of its component elements, the nanovector design is susceptible to improvement and adaptation to new targets such as different *Plasmodium* species or infected cells other than the erythrocyte. Of particular interest here is the targeting of the transmission stages that allow transfer of the parasite between human and mosquito and *vice-versa*, which represent the weakest spots in the life cycle of the pathogen.^17^ Heparin and HS are targets for the circumsporozoite protein in sporozoite attachment to hepatocytes during the primary stage of malaria infection in the liver.^18^ CS proteoglycans in the mosquito midgut and synthetic CS mimetics have been described to bind *Plasmodium* ookinetes as an essential step of host epithelial cell invasion,^19^, ^20^ whereas ookinete-secreted proteins have significant binding to heparin.^21^ This body of accumulated evidence suggests that GAGs might be adequate to target antimalarial-loaded nanovectors to *Plasmodium* mosquito stages, either through a direct entry into ookinetes and sporozoites, or indirectly through delivery to pRBCs for those pRBCs that will eventually differentiate into gametocytes.

Here we have explored whether the heparin- and antibody-mediated targeting of drug-containing liposomes to pRBCs could be adapted in a straightforward way to other GAGs as targeting molecules, to different *Plasmodium* stages as target cells, and to new nanoparticle and drug types.

## Methods

### Materials

Except where otherwise indicated, reactions were performed at room temperature (20 °C), reagents were purchased from Sigma-Aldrich Corporation (St. Louis, MO, USA), and cultures of the *P. falciparum* 3D7 strain have been used. The lipids (all ≥99% purity according to thin layer chromatography analysis) 1,2-dioleoyl-*sn*-glycero-3-phosphocholine (DOPC), L-α-phosphatidylethanolamine (PE), 1,2-dipalmitoyl-*sn*-glycero-3-phosphoethanolamine-N-(4-(p-maleimidophenyl)butyramide (MPB-PE), 1,2-dioleoyl-*sn*-glycero-3-phosphoethanolamine-N(lissamine rhodamine B sulfonyl) (DOPE-Rho), and 1,2-dioleoyl-3-trimethylammonium-propane (DOTAP) were purchased from Avanti Polar Lipids Inc. (Alabaster, AL, USA).

### Liposome preparation

Established protocols were used for liposome^22^ and immunoliposome preparation.^23^ In Supplementary Video 1 can be seen an example of a pRBC culture treated with rhodamine-labeled immunoliposomes targeted to pRBCs as described elsewhere.^23^ Liposome size was determined by dynamic light scattering using a Zetasizer NanoZS90 (Malvern Ltd., Malvern, UK).

### Preparation of primaquine-containing liposomes functionalized with covalently bound heparin

The antimalarial drug primaquine (PQ) was encapsulated in DOTAP-containing liposomes (DOPC:PE:cholesterol:DOTAP, 46:30:20:4) by dissolving it at 1.2 mM in the PBS buffer used to hydrate the lipids, removing non-encapsulated drug by ultracentrifugation (150,000×*g*, 1 h, 4 °C). To crosslink the amine groups present in the liposomes with the carboxyl groups of heparin (sodium salt from porcine intestinal mucosa, 13 kDa mean molecular mass) or its hexa- and octasaccharide fragments (Iduron, Cheshire, UK), the polymers were first dissolved at 1 mg/mL in MES activation buffer (0.1 M 2-(N-morpholino)ethane sulfonic acid, 0.5 M NaCl, pH 5.0). Final concentrations of 2 mM N-(3-dimethylaminopropyl)-N′-ethylcarbodiimide hydrochloride (EDC, Fluka) and 5 mM N-hydroxysuccinimide (NHS, Fluka) were added to the activated heparin solution. To obtain the desired heparin:liposome ratios, after 15 min the corresponding heparin solution and liposome suspension volumes in PBS buffer were mixed and incubated for 2 h with gentle stirring. To remove unbound heparin, liposomes were pelleted by ultracentrifugation (150,000×*g*, 1.5 h, 4 °C), and taken up in 10 pellet volumes of PBS immediately before addition to pRBC cultures with a further *ca.* 20-fold dilution (to obtain 3 μM final PQ concentration in the culture). For the quantification of encapsulated PQ, a lipid extraction of the liposomes was performed. Briefly, following ultracentrifugation the liposome pellet was treated with methanol:chloroform:0.1 M HCl (1.8:2:1) and after phase separation the PQ content in the upper water–methanol phase was determined by measuring A_320_ against a calibration curve of known PQ concentrations. *In vitro* coagulation tests of heparin-containing liposomes were done as previously described.^16^ Heparin concentration was determined by the Alcian Blue method.^24^

### Chitosan nanoparticle synthesis

Chitosan nanoparticles were prepared by a coacervation method described elsewhere.^25^ Briefly, 0.5 g chitosan (low molecular weight, 75-85% deacetylated, Aldrich Ref. 448869) was dissolved in 50 mL of an aqueous solution of 2% v/v acetic acid containing 1% w/v Pluronic® F-68. About 12.5 mL of a 20% w/v sodium sulfate solution was added dropwise (2.5 mL/min) to the chitosan solution under mechanical stirring (1200 rpm) for 1 h to obtain a suspension of chitosan nanoparticles. The colloidal suspension was then subjected to a cleaning procedure that included repeated cycles of centrifugation (40 min, 14,000×*g*; Centrikon T-124 high-speed centrifuge, Kontron, Paris, France) and re-dispersion in water, until the conductivity of the supernatant was ≤10 μS/cm. Particle size was determined by photon correlation spectroscopy using a Malvern 4700 analyzer (Malvern Ltd). The measurement was made under a 60° scattering angle of the aqueous nanoparticle suspensions (0.1%, w/v). The electrophoretic mobility measurements were performed in 0.1% (w/v) aqueous suspensions of nanoparticles in 1 mM KNO_3_, pH 7, using a Malvern Zetasizer 2000 electrophoresis device (Malvern Ltd), under mechanical stirring (50 rpm) at 25 °C. The electrophoretic mobility was converted into zeta potential (ζ, mV) values as described by O′Brien and White.^26^

### Determination of chitosan–heparin interaction

Isothermal titration calorimetry (ITC) measurements were performed with a VP-ITC microcalorimeter following established protocols.^16^ For fluorescence determinations, chitosan nanoparticles (5 mg/mL) and heparin labeled with fluorescein isothiocyanate (heparin-FITC, Life Technologies) were mixed 10:1 w/w and incubated for 90 min with gentle orbital mixing. After a centrifuge step (100,000×*g*, 1 h, 4 °C) to remove unbound heparin, the pellet was taken up in PBS, its fluorescence measured (λ_ex_/_em_: 488/525 nm), and the corresponding concentration determined against a standard linear regression of known FITC concentrations. The fluorescence of the supernatant was also measured to confirm that it contained the fraction of heparin not associated with the nanoparticles.

### Plasmodium falciparum cell culture

The *P. falciparum* strains 3D7 and CS2 (MRA-96, obtained through the MR4 as part of the BEI Resources Repository, NIAID, NIH, deposited by SJ Rogerson) were grown *in vitro* in group B human RBCs using previously described conditions.^27^

### Plasmodium berghei ookinete culture and targeting assay

Ookinete culture medium consisted of 16.4 g/L Roswell Park Memorial Institute (RPMI) medium supplemented with 2% w/v NaHCO_3_, 0.05% w/v hypoxanthine, 100 μM xanthurenic acid, 50 U/mL penicillin, 50 μg/mL streptomycin (Invitrogen), 25 mM HEPES, pH 7.4. Complete medium was prepared just before use by supplementing with heat-inactivated fetal bovine serum (FBS, Invitrogen) to a final concentration of 20%. Six days prior to performing the targeting assay, a mouse was treated intraperitoneally with 10 μg/mL phenylhydrazine (PHZ) to induce reticulocytosis. Three days after PHZ treatment the mouse was inoculated by intraperitoneal injection of 200 μL of blood containing *ca.* 5 × 10^7^ *P. berghei* mCherry (a kind gift from Dr. D. Vlachou) pRBCs extracted by cardiac puncture from a donor mouse that had been infected intraperitoneally 3 days before with 200 μL of a cryopreserved *P. berghei* suspension just thawed. Three days later, 1 mL of infected blood was collected by cardiac puncture onto 30 mL ookinete medium, and incubated for 24 h at 19-21 °C with 70-80% relative humidity. For ookinete targeting assays, 100 μL of 0.25 mg/mL heparin-FITC was added to 100 μL of culture and incubated in the dark for 90 min under orbital stirring (300 rpm). The samples were centrifuged for 1.5 min at 800×*g* and washed 3× with PBS. Fixed cell slides were prepared by adding 0.5 μL FBS to 0.5 μL pellet and by fixing the smear with 4% paraformaldehyde for 15 min. After performing 3 washing steps with PBS, slides were mounted with Vectashield® 4′6-diamino-2-phenylindole (DAPI)-containing media (Vector Laboratories, UK). All work involving laboratory animals was performed with humane care in accordance with EU regulations (EU Directive 86/609/EEC) and with the terms of the United Kingdom Animals (Scientific Procedures) Act (PPL 70/8788), and was approved by the Imperial College Ethical Review Committee.

### Microscopy

Existing protocols were used for the fluorescent labeling of CSA,^28^ fluorescence confocal microscopy^16^ and cryo-transmission electron microscopy^29^ sample imaging. Details of these techniques are provided in the Supplementary Materials.

### Force spectroscopy

Binding forces between CSA and pRBCs infected with the *P. falciparum* CS2 strain were measured by atomic force microscope (AFM) single-molecule force spectroscopy (SMFS) essentially as described elsewhere.^14^ A complete protocol is provided in the Supplementary Materials.

### Statistical analysis

Data are presented as the mean ± standard deviation of at least three independent experiments, and the corresponding standard deviations in histograms are represented by error bars. The parametric Student's *t* test was used to compare two independent groups when data followed a Gaussian distribution, and differences were considered significant when *P* ≤ 0.05. Percentages of viability were obtained using non-treated cells as control of survival and IC50 values were calculated by nonlinear regression with an inhibitory dose–response model using GraphPad Prism5 software (95% confidence interval). Concentrations were transformed using natural log for linear regression, and regression models were adjusted for the assayed replicates.

## Results

### Use of targeted liposomes for the delivery of antimalarial lipids to Plasmodium

**Figure 1:**
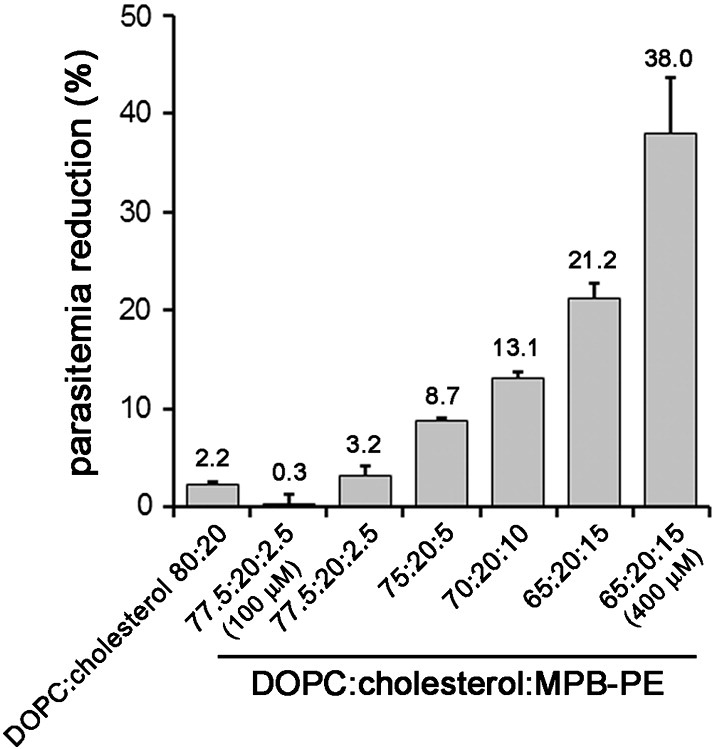
Determination of the concentration-dependent effect of the lipid MPB-PE on the *in vitro* growth of *P. falciparum*. Concentrations of the liposome formulations in the cultures were 200 μM lipid except where otherwise indicated.

**Figure 2:**
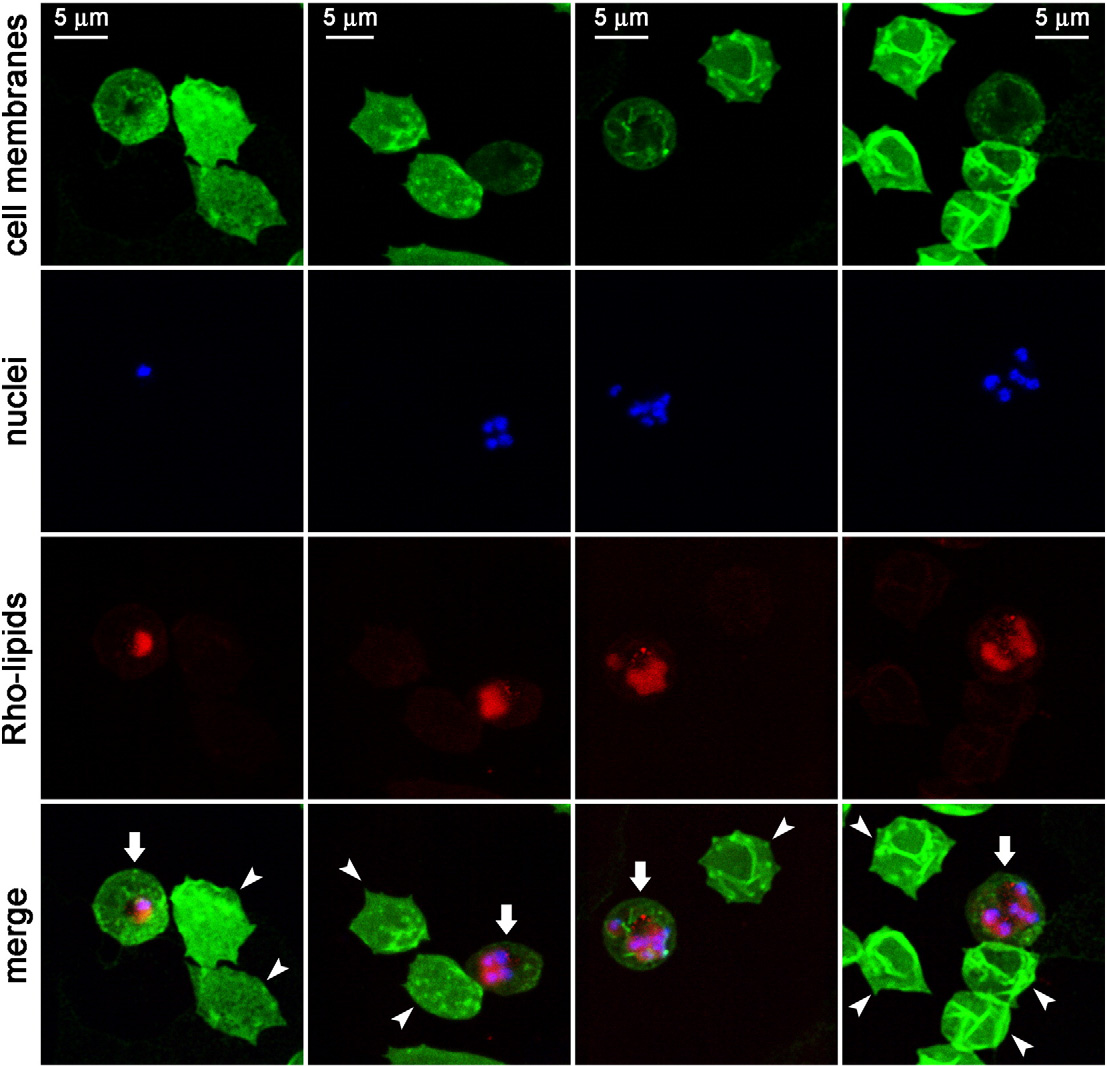
Fluorescence confocal microscopy analysis of the fate of Rho-labeled lipids incorporated in the formulation of pRBC-targeted immunoliposomes added to living *P. falciparum* cultures and incubated for 90 min before proceeding to sample processing. Arrows indicate pRBCs and arrowheads RBCs.

**Figure 3:**
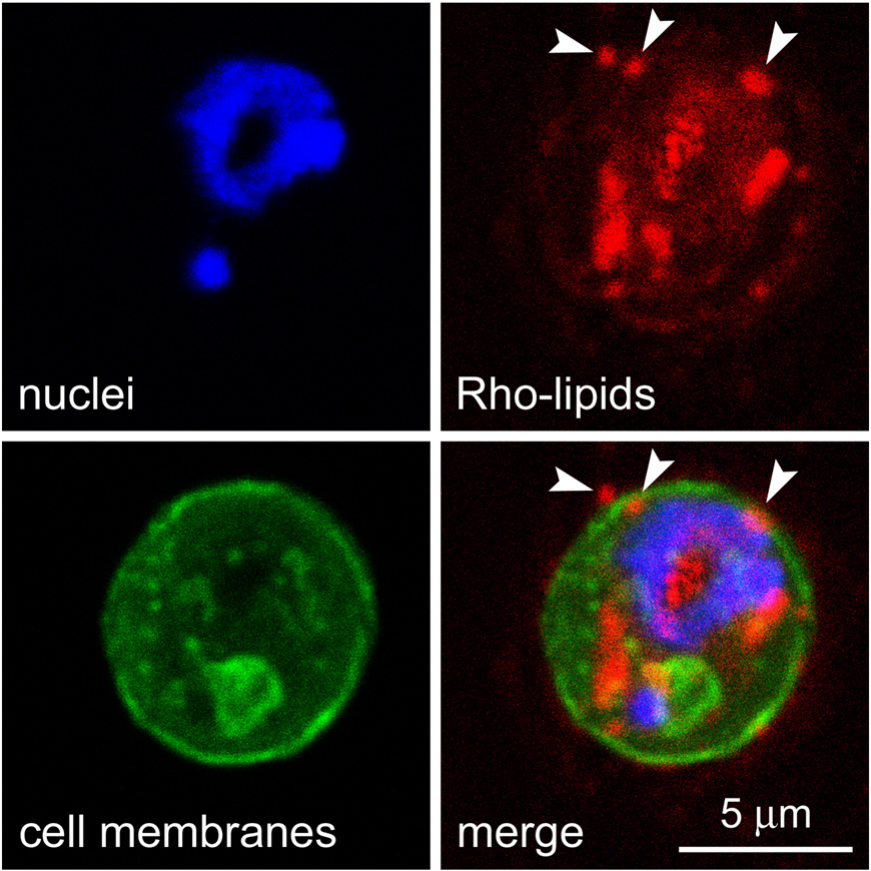
Fluorescence confocal microscopy analysis of a pRBC showing the subcellular distribution of Rho-labeled lipids incorporated in the formulation of pRBC-targeted immunoliposomes added to living *P. falciparum* cultures. Arrowheads indicate structures compatible with plasma membrane-liposome merging events.

Preliminary data suggesting antimalarial activity of certain lipids^23^ led us to explore this observation in more detail. The lipid MPB-PE, used for the covalent crosslinking to liposomes of antibodies through thioether bonds, exhibited significant concentration-dependent inhibition of the *in vitro* growth of *P. falciparum* when incorporated in the formulation of liposomes (Figure 1). This antiparasitic effect suggested that, upon random interactions of liposomes with pRBCs, lipids entered the cell and reached the pathogen. To explore whether such process occurred through whole liposome entry or was mediated by transfer phenomena between the apposed lipid bilayers of liposomes and pRBCs, we performed confocal fluorescence microscopy analysis of pRBC-targeted immunoliposomes containing in their formulation 7% of the rhodamine-tagged lipid DOPE-Rho. Specific pRBC targeting was achieved as previously described^23^ through functionalization of the liposomes with the monoclonal antibody BM1234 raised against the *P. falciparum*-expressed membrane-associated histidine-rich protein 1.^8^ The results obtained with *P. falciparum* cultures containing RBCs and 5% pRBCs (Figure 2) showed that targeted liposome-administered lipids were specifically delivered to pRBCs and after 90 min of incubation colocalized with intracellular parasites. The observation of diffuse fluorescence and the lack of punctate patterns characteristic of whole intact liposomes^8^ suggests that upon contact with the pRBC plasma membrane, liposomes fused with the cell and their constituent lipids were incorporated by the growing parasites. Whole liposome entry into pRBCs might theoretically occur through the reported tubulovesicular network induced by *Plasmodium* during its intraerythrocytic growth,^30^ which extends from the parasitophorous vacuole membrane and connects the intracellular parasite with the host RBC surface. However, this confers to the pRBC the capacity of internalizing a wide range of particles up to diameters of only 70 nm,^30^, ^31^ well below the mean size of the liposomes used here (>140 nm, Figure S1). Higher resolution images of cells prepared at earlier stages in the drug delivery process revealed phenomena consistent with the interaction of liposomes with pRBCs immediately before or just after their constituent lipids are incorporated into the cell plasma membrane (Figure 3).

### Antimalarial activity of drug-loaded liposomes targeted with covalently bound heparin

**Figure 4:**
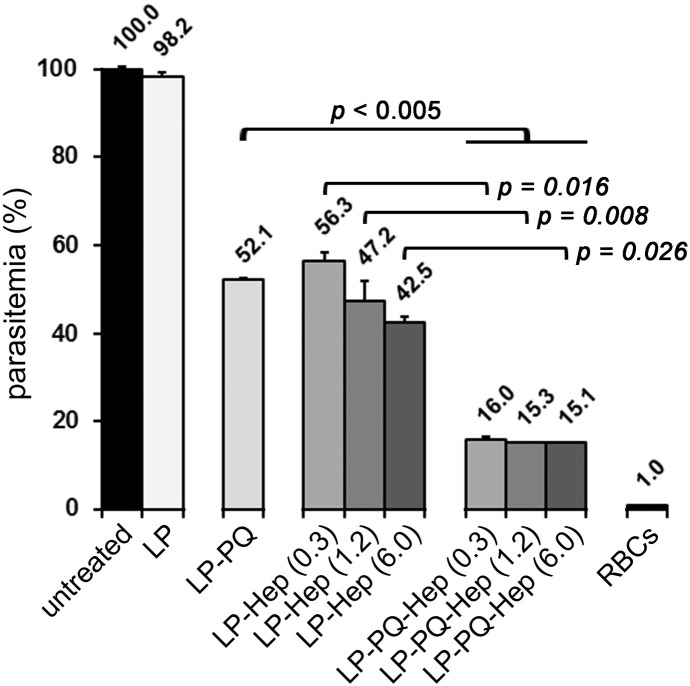
Antimalarial activity and targeting capacity of different amounts of heparin covalently bound to primaquine-containing liposomes (LP-PQ-Hep). Controls include plain liposomes (LP), heparin-free, primaquine-containing liposomes (LP-PQ) and primaquine-free liposomes targeted with covalently-bound heparin (LP-Hep). PQ concentration in the pRBC culture was 3 μM for all samples. In parentheses are indicated the determined μg/mL of liposome-bound heparin present in *P. falciparum* cultures.

The dual activity of heparin as an antimalarial drug and as the pRBC targeting element has been proposed as a promising new avenue for future malaria therapies.^32^ However, existing models contain electrostatically bound heparin^16^ that is prone to peel off from liposome surfaces while in the blood circulation, incurring the risk of anticoagulation and internal bleeding. To explore strategies that could minimize these adverse effects, we have modified our previous design to incorporate covalently bound heparin on primaquine (PQ)-loaded liposomes. PQ was selected because its high IC50 for *in vitro P. falciparum* cultures allowed an immediate and easy sample concentration determination, but also for reasons regarding current needs in antimalarial chemotherapy. In patients with glucose-6-phosphate dehydrogenase (G6PD) deficiency PQ generally induces RBC oxidative damage that eventually results in hemolytic anemia which may be severe.^33^, ^34^ Such toxicological concerns have led to restrictions in the use of this drug since the incidence of G6PD genetic anomaly is particularly high in areas where malaria is endemic,^35^ a situation that calls for new methods addressed to the targeted delivery of PQ active species to pRBCs. The new liposome prototype exhibited an additive effect whereby PQ-loaded liposomes had a significantly improved antimalarial activity when targeted with covalently bound heparin (Figure 4), suggesting the double role of this GAG as drug and targeting molecule. The anticoagulant activity of heparin covalently bound to liposomes (Table 1) was found to be significantly smaller than similar amounts electrostatically bound,^16^ in agreement with previous evidence of non-anticoagulant activity of heparin when covalently immobilized on a substrate.^36^

**Figure 5:**
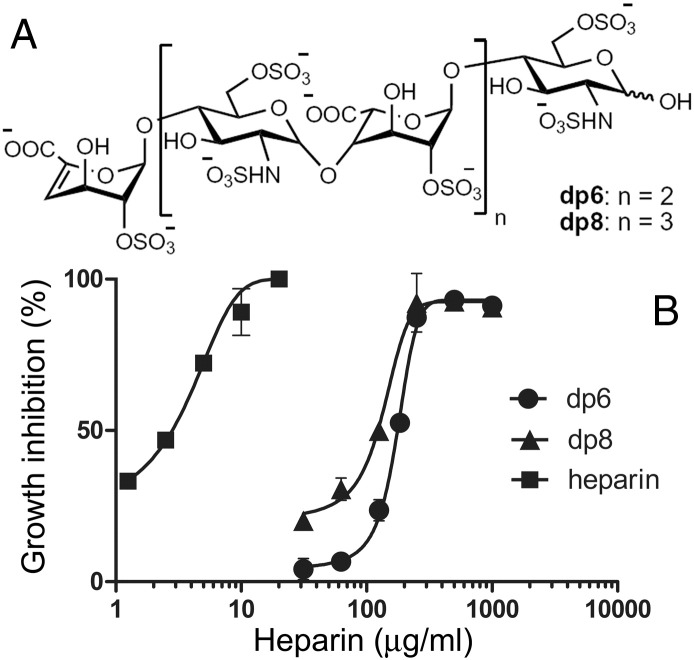
*In vitro* antimalarial activity of heparin fragments compared to that of heparin. **(A)** Chemical structure of the hexa- and octasaccharides dp6 and dp8. **(B)***P. falciparum* growth inhibition assay.

Depolymerized heparin lacking anticlotting activity had been found to disrupt rosette formation and pRBC cytoadherence *in vitro* and *in vivo* in animal models and in fresh parasite isolates.^37^, ^38^ Shorter heparin fragments consisting of hexa- and octasaccharides (dp6 and dp8; Figure 5, *A*) having insignificant anticoagulant activity^39^ exhibited a much smaller antimalarial activity *in vitro* than the native polymer, with respective IC50s of 174 and 134 μg/mL, compared to around 4 μg/mL for heparin (Figure 5, *B*). Neither heparin oligosaccharide covalently bound to PQ-loaded liposomes improved the activity of the liposomized drug (data not shown), suggesting that also the pRBC targeting capacity of heparin is significantly lost upon depolymerization.

### Functionalization of chitosan nanoparticles with heparin

**Figure 6:**
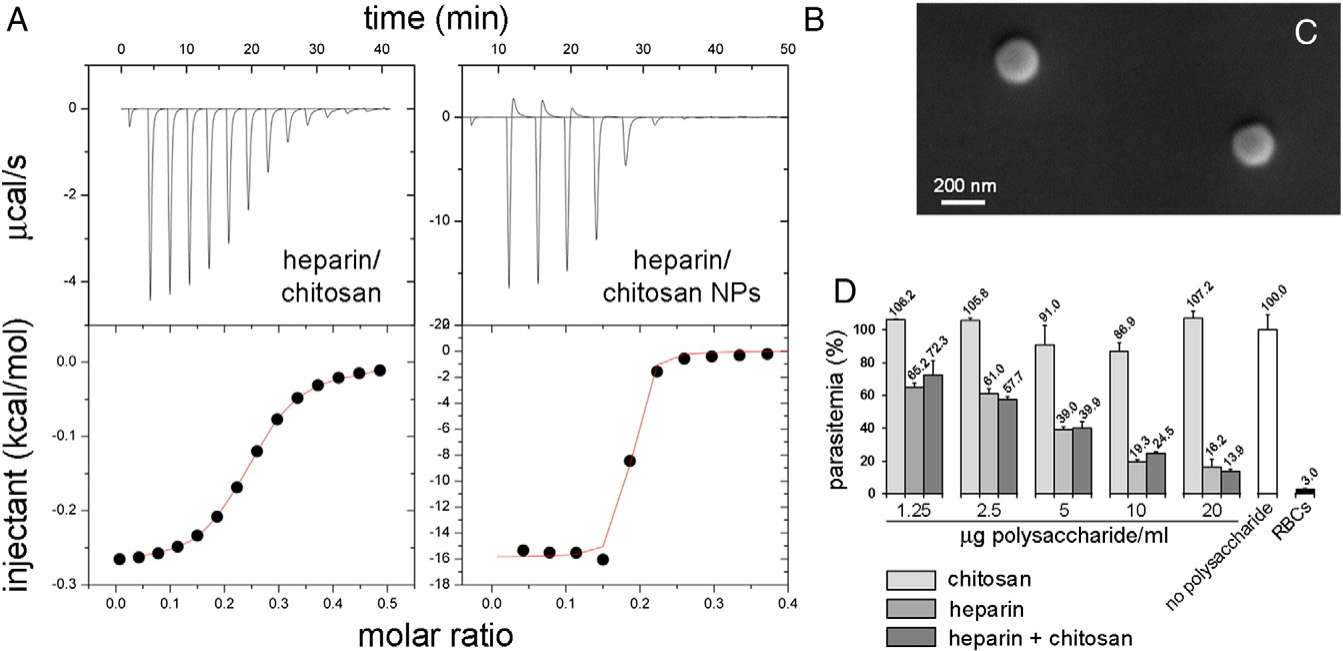
Study of the interaction between heparin and chitosan. **(A)** Representative data from an ITC experiment in which heparin was titrated into the reaction cell containing chitosan. Aliquots of a 0.05 mM heparin solution were injected to a 0.01 mM chitosan solution in the ITC cell. The area underneath each injection peak (top panel) is equal to the total heat released for that injection. When this integrated heat is plotted against the respective molar ratios in the reaction cell, a complete binding isotherm for the interaction is obtained (bottom panel). **(B)** Representative data from an ITC experiment in which aliquots of a 1 mg/mL heparin solution were injected into the reaction cell containing 0.1 mg/mL chitosan nanoparticles (NPs). **(C)** Scanning electron microscopy image of the chitosan nanoparticles used. **(D)** Effect on the antimalarial activity of heparin of its interaction with chitosan. In heparin + chitosan samples the plotted concentration refers to only one of the polysaccharides (the other being present in an equal amount).

The highly specific binding of heparin to pRBCs *vs.* RBCs^14^ prompted us to explore its capacity as a targeting agent of nanoparticles other than liposomes. The electrostatic interaction of heparin with positively charged nanocapsules has been explored as a proof of concept with the objective of designing the simplest functionalization strategy. ITC was used to analyze the interaction of heparin with the cationic polymer chitosan (Figure 6), whose biocompatibility makes it a preferred material for biomedical applications.^40^, ^41^, ^42^ A complete sigmoidal exothermic binding isotherm for the interaction heparin–chitosan was observed, with a 50% saturation obtained at a molar ratio chitosan:heparin of 0.25 and a binding constant of 7.9 ± 0.6 × 10^3^ M^−1^ fitted to a model of identical binding sites (Figure 6, *A*). Chitosan nanoparticles were synthesized with an average diameter of 140 ± 30 nm (Figure 6, *C*) and a positive surface charge (zeta potential, ζ, of 18 ± 4 mV at 25 °C and pH 7.0). When heparin was added to chitosan nanoparticles a strong cooperative effect was observed with a 3 orders of magnitude increase for the binding constant (4.6 ± 2.6 × 10^6^ M^−1^) fitted to the same binding model (Figure 6, *B*). Likely, the association of multiple chitosan molecules in a nanoparticle favored the cooperative binding of heparin to adjacent chitosan chains following an initial interaction. In pull-down assays where 0.5 mg/mL heparin-FITC was mixed with chitosan nanoparticles at a 1:10 w/w ratio, 93% of heparin was found to be bound to the pelleted nanoparticles (data not shown). Cryo-transmission electron microscopy analysis indicated that heparin was not tightly bound to chitosan nanoparticles, but it rather formed a loose network around them (Figure S2). According to *in vitro P. falciparum* growth inhibition assays the interaction of heparin with chitosan did not affect its antimalarial activity (Figure 6, *D*).

### Targeting of heparin to Plasmodium stages in the mosquito vector

**Figure 7:**
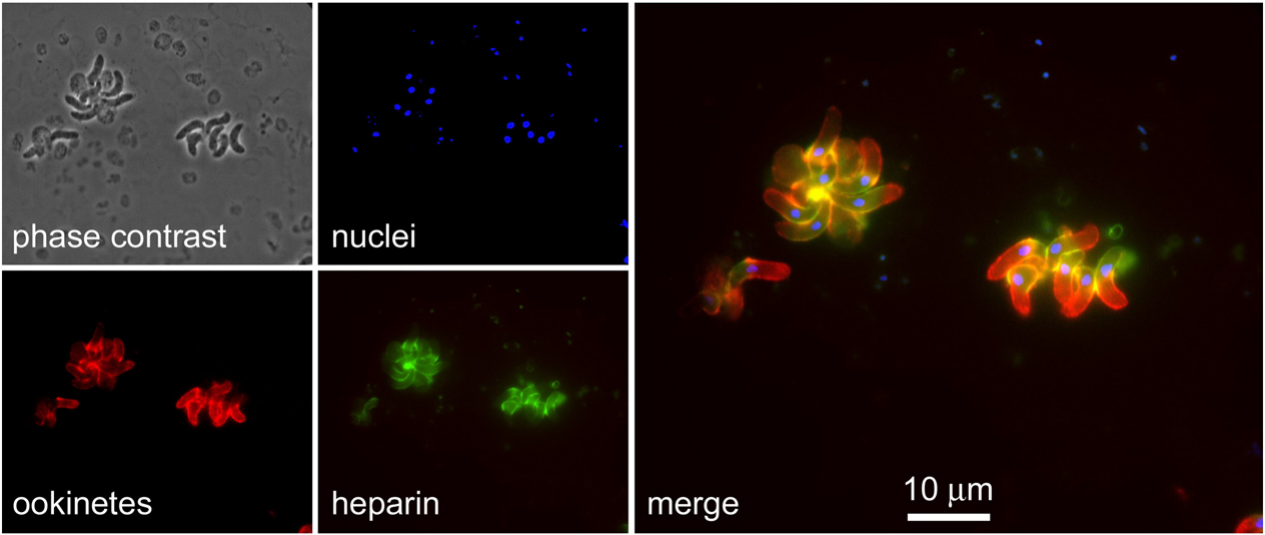
Fluorescence confocal microscopy analysis of the binding of heparin-FITC to *P. berghei* ookinetes *in vitro*. Ookinete fluorescence is shown by mCherry and parasite nuclei were stained with DAPI.

The straightforward binding of heparin to chitosan results in nanoparticles likely to be innocuous for insects given the endogenous nature of chitosan in these animals and the expected imperviousness of mosquitoes to the presence of blood anticlotting agents. This stimulated us to study the targeting capacity of heparin towards the *Plasmodium* stages in *Anopheles*. Fluorescently labeled heparin-FITC added to preparations containing *Plasmodium* gametocytes, ookinetes, oocysts or sporozoites was observed to bind only to ookinetes (Figures 7 and S3). Here we have followed the available protocols for ookinete *in vitro* production which use the murine malaria parasite *P. berghei*, although our results are in agreement with previous data reporting on *P. falciparum* ookinete proteins binding heparin,^21^ chondroitin sulfate GAGs,^19^ and GAG mimetics.^20^

### Use of CSA for the targeting of pRBCs

**Figure 8:**
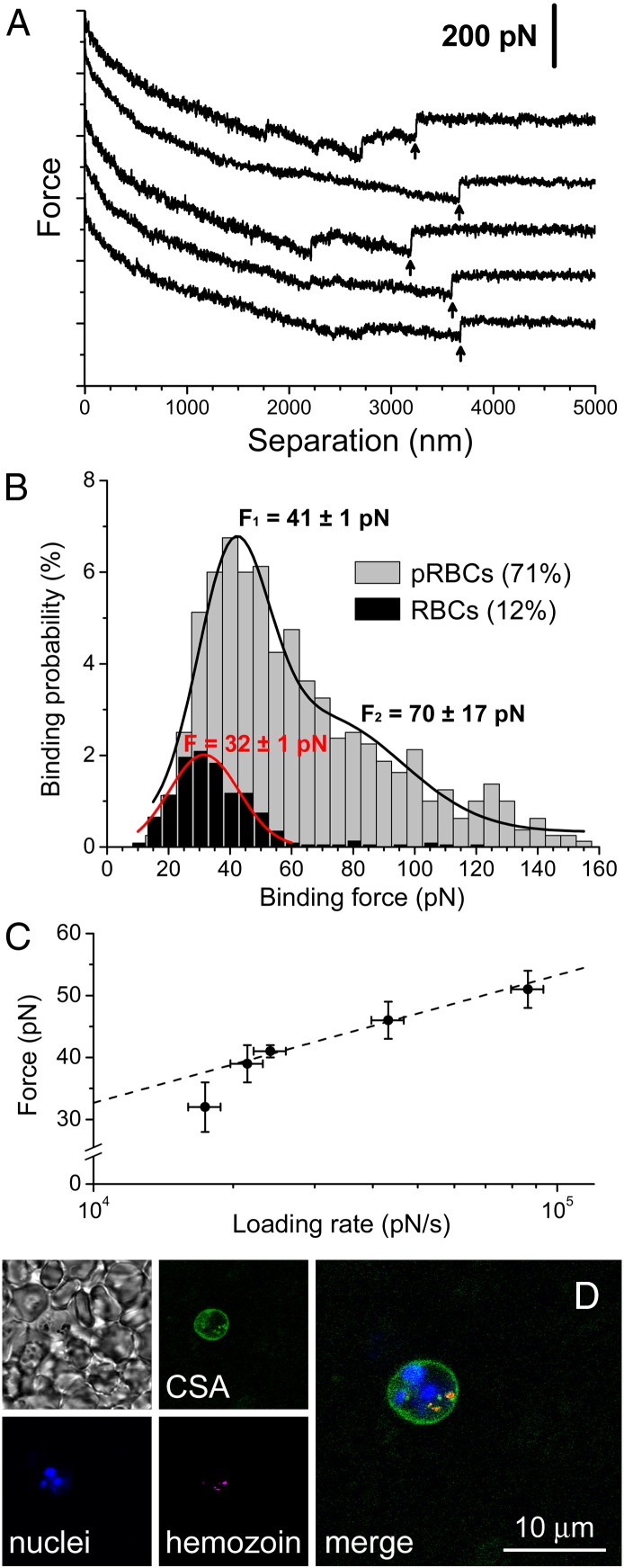
Study of CSA binding to erythrocytes. **(A)** Typical AFM-SMFS force curves obtained when retracting CSA-functionalized cantilever tips from pRBCs. Arrows indicate individual CSA-pRBC unbinding events. For the sake of clarity, the force curves were shifted vertically to avoid overlapping. **(B)** Representative force histograms for the binding of CSA to pRBCs (gray) and RBCs (black) at a loading rate of 24 nN s^−1^. Force histograms were fitted to a Gaussian (RBC) or a 2-peak Gaussian function (pRBC). **(C)** Average binding forces between CSA and pRBCs at different loading rates. The dashed line corresponds to the linear fit of the experimental data. **(D)** Fluorescence confocal microscopy analysis of the *in vitro* binding of fluorescent CSA to living pRBCs infected with the *P. falciparum* CS2 strain. The phase contrast image in the upper left panel evidences the presence of several non-infected RBCs in the microscope field. As a pRBC marker, hemozoin crystal reflection is shown in red in addition to DNA stain.

As discussed above, the potential use of heparin as drug in malaria therapy^15^, ^43^, ^44^, ^45^ has been hindered by its anticlotting properties,^46^ but heparin-related polysaccharides exist which are known to have little anticoagulating activity. One such polysaccharide is CSA, which lacks antimalarial activity^47^ but whose pRBC targeting capacity has barely been explored. We have used AFM-SMFS to measure the binding forces between CSA and pRBCs or non-infected RBCs deposited on poly-L-lysine-coated glass slides. CSA molecules were immobilized on the tip of cantilevers used as force sensors, which were approached to the adsorbed erythrocytes and retracted from them after contact in order to obtain a force curve. Single-molecule CSA-pRBC adhesion forces in PBS were evaluated from the unbinding events found in *ca.* 50% to 71% of total retraction force curves (Figure 8, *A*). As the CSA-coated tip withdrew, a decompression and stretching of the pRBC were observed in the retraction force curves for distances up to 4 μm, which was followed by a vertical jump (arrows in Figure 8, *A*) corresponding to the detachment of the tip from the cell membrane. A flat baseline was finally reached, indicating no interaction between cell and tip after their complete separation. A representative histogram for CSA-pRBC adhesion (Figure 8, *B*) shows an average binding force of 41 ± 1 pN for the main peak. A second, smaller peak at 70 ± 17 pN, and possibly a third one at about 120 pN (not included in the fit), could correspond to the simultaneous unbinding of 2 and 3 interacting groups on the same or different CSA molecules, respectively. In dynamic force spectroscopy assays performed at different loading rates, binding forces between 32 and 51 pN were calculated for the main peaks of the histograms obtained (Figure 8, *C*). A linear relation between binding force and logarithm of loading rate was observed, in agreement with the predictions from Bell–Evans model for binary interactions.^48^, ^49^ Control experiments with non-infected RBCs showed adhesion to CSA in only a small proportion (9%-12%) of the retraction force curves, with generally smaller binding forces than for pRBCs (*e.g.* 32 ± 1 pN for the representative histogram in Figure 8, *B*). This specificity of adhesion was confirmed in fluorescence confocal microscopy assays (Figure 8, *D*).

The adhesion between pRBCs infected by the CSA-binding *P. falciparum* FCR3-CSA strain and Chinese hamster ovary (CHO) cells expressing CSA on their surface had been explored by AFM force spectroscopy,^50^ yielding a mean rupture force of 43 pN, similar to that obtained here using purified CSA. Because CSA interaction with pRBCs has been described to occur through the binding to PfEMP1 on erythrocyte surfaces, the adhesive force between both cell types had been assigned entirely to the CSA-PfEMP1 association.^50^ The binding of CSA on the AFM cantilever to pRBCs could not be inhibited by the presence of 500 μg CSA/mL in solution (Figure S4), whereas pRBC-CHO adhesion had been shown to be significantly blocked (*ca.* 90% inhibition) by 100 μg CSA/mL.^51^ This discrepancy can likely be explained by invoking the much larger CSA concentration on AFM cantilevers in SMFS assays than on CHO cell surfaces.

## Discussion

Despite the lack of economic incentives for research in nanomedicine applications to malaria a number of liposome- and polymer-based nanocarriers engineered for the targeted delivery of antimalarial drugs have been developed.^5^, ^6^, ^8^, ^16^, ^23^, ^29^, ^52^, ^53^ Although successful efforts have been made to obtain new nanostructures having affordable synthesis costs while still exhibiting good performance in lowering the IC50 of drugs,^16^, ^29^ new approaches are required to further optimize these scarce resources. The implementation of novel delivery approaches is less expensive than finding new antimalarial drugs and may improve the rate of release of current and future compounds.^54^ The three elements that constitute a targeted therapeutic nanovector (nanocapsule, targeting molecule and the drug itself) can be exchanged, as if they were LEGO blocks, to obtain new structures better suited to each particular situation.

The data presented here allow us to propose several combinations of nanovector parts that could be adapted to new antimalarial strategies: (i) liposomes formulated with antimalarial lipids and targeted with covalently bound heparin could carry the active agents in their bilayer membranes with little leaking before reaching their target site and with low hemorrhagic risk. Although liposomes are not adequate for the oral formulations currently required to treat malaria in endemic areas, intravenous administration of drugs might be a useful approach in a future eradication scenario where the last cases caused by hyper-resistant parasite strains will be amenable to treatment with sophisticated, targeted liposomal nanocarriers. Liposomes have a long record of proven biocompatibility and their lipid formulation can be adapted to obtain either fast or slow drug release,^8^ which makes them adaptable to carrying antimalarial drugs with diverse pharmacokinetic profiles. (ii) Since resistance of *Plasmodium* to heparin has not been shown so far,^55^ heparin-based targeting will predictably be more long-lasting than pRBC recognition relying on antibodies, which typically are raised against highly variable exposed antigens whose expression is constantly modified by successive generations of the parasite.^56^ The specific binding of CSA to pRBCs infected by the *P. falciparum* CS2 strain, which sequester in the maternal circulation of the placenta,^57^ suggests that future nanovectors functionalized with CSA can be foreseen to be adapted to target drugs to pRBCs for the treatment of placental malaria. Such nanocarriers will bypass the concerns discussed above regarding the hemorrhagic risks of administering heparin to humans, since CSA has been shown to lack anticoagulant activity.^47^ (iii) Finally, the engineering of antimalarial nanomedicines designed to be delivered to mosquitoes and targeted to *Plasmodium* stages exclusive to the insect might spectacularly reduce costs because the clinical trials otherwise required for therapies to be administered to people could be significantly simplified. Strategies that control malaria using direct action against *Anopheles* are not new, but most of them aim at eliminating the vector, either by killing it with pesticides^58^ or through the release of sterile males.^59^, ^60^ Since eradicating an insect species might have as a consequence unpredictable disruptions of ecosystems with potential undesirable side effects (*e.g.* crop failure if pollinators were inadvertently affected), mosquito-friendly antimalarial strategies should be favored whenever possible. Thus, administration of drugs to mosquitoes to free them of malaria with the objective of blocking transmission of the disease is a realistic alternative worth exploring.

## Figures and Tables

**Table 1:**
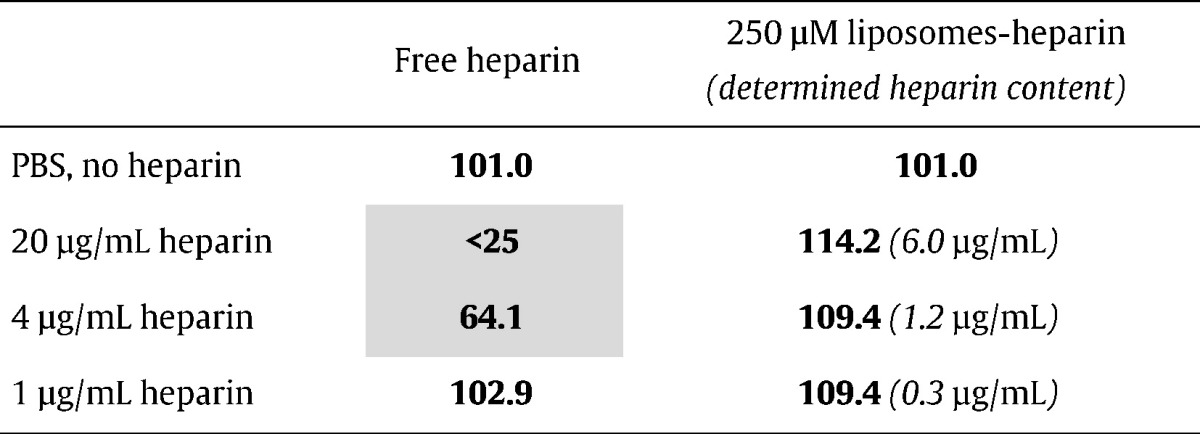
*In vitro* coagulation test of different heparin concentrations, free or covalently conjugated to liposomes.

Liposome preparations initially containing the same heparin amounts as liposome-free samples were ultracentrifuged to remove unbound heparin and the new heparin content was experimentally determined; the values indicated in parentheses correspond to actual heparin concentrations in *P.falciparum* cultures that result from adjusting the volume of liposome suspension added to obtain a final 3 μM PQ. Coagulation capacity is expressed as a percentage relative to the value obtained with standard human plasma. Shadowed in gray are indicated those samples with anticoagulant activity.

## Appendix A Supplementary data

Supplementary Video 1 Living *P. falciparum* cultures with mature stages of the parasite were incubated in glass bottom dishes (MatTek Corporation) in the presence of immunoliposomes containing in their formulation 1% DOPE-Rho (red fluorescence; 400 μM total lipid content in the dish) targeted to pRBCs (identified by Hoechst 33342 blue fluorescence). Phase contrast imaging is used to show the erythrocytes during the 12 h of duration of the assay. Supplementary Video 1
