# Supplementary figures and images for: Adaptation of targeted nanocarriers to changing requirements in antimalarial drug delivery
Source: Nanomedicine. 2017 Feb;13(2):515–25. doi: 10.1016/j.nano.2016.09.010 (PMC5332526; doi:10.1016/j.nano.2016.09.010)

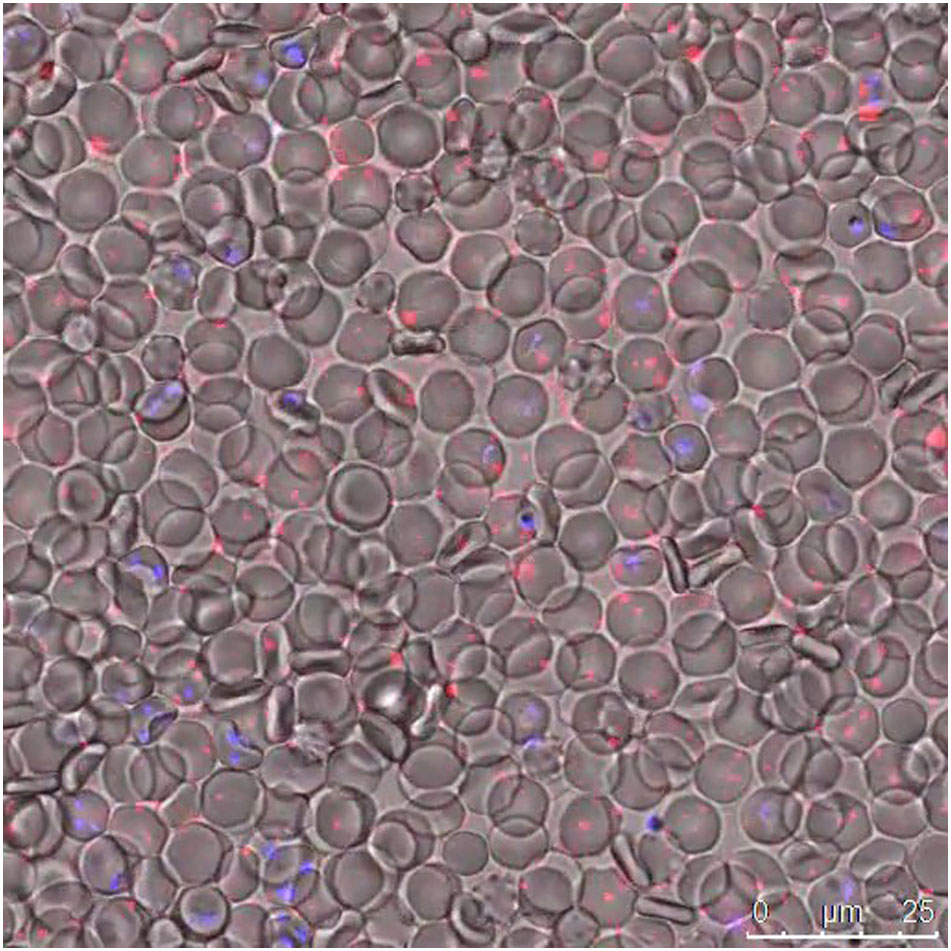

Supplement: Supplementary Video 1 — Living P. falciparum cultures with mature stages of the parasite were incubated in glass bottom dishes (MatTek Corporation) in the presence of immunoliposomes containing in their formulation 1% DOPE-Rho (red fluorescence; 400 μM total lipid content in the dish) targeted to pRBCs (identified by Hoechst 33342 blue fluorescence). Phase contrast imaging is used to show the erythrocytes during the 12 h of duration of the assay. [file mmc1.jpg]
