# Supplementary material for: Adaptation of targeted nanocarriers to changing requirements in antimalarial drug delivery
Source: Nanomedicine. 2017 Feb;13(2):515–25. doi: 10.1016/j.nano.2016.09.010 (PMC5332526; doi:10.1016/j.nano.2016.09.010)
Supplement: Supplementary materials [file mmc2.pdf]

Adaptation of targeted nanocarriers to changing requirements in antimalarial drug delivery

Joana Marques, PhD[a,b,c,1], Juan José Valle-Delgado, PhD[a,b,c,2], Patricia Urbán, PhD[a,b,c,3], Elisabet Baró, MSc[a,b,c], Rafel Prohens, PhD[d], Alfredo Mayor, PhD[b], Pau Cisteró, BSc[b], Michael Delves, PhD[e], Robert E. Sinden, DSc, FMedSci[e], Christian Grandfils, PhD[f], José L. de Paz, PhD[g], José A. García-Salcedo, PhD[h], and Xavier Fernàndez-Busquets, PhD[a,b,c,*]

[a]*Nanomalaria Group, Institute for Bioengineering of Catalonia (IBEC), Baldiri Reixac 10-12, ES-08028 Barcelona, Spain*
[b]*Barcelona Institute for Global Health (ISGlobal), Barcelona Center for International Health Research (CRESIB, Hospital Clínic-Universitat de Barcelona), Rosselló 149-153, ES-08036 Barcelona, Spain*
[c]*Nanoscience and Nanotechnology Institute (IN2UB), University of Barcelona, Martí i Franquès 1, ES-08028 Barcelona, Spain*
[d]*Unitat de Polimorfisme i Calorimetria, Centres Científics i Tecnològics, Universitat de Barcelona, Baldiri Reixac 10, ES-08028 Barcelona, Spain*
[e]*Department of Life Sciences, Imperial College, South Kensington, London, SW7 2AZ, UK*
[f]*Interfacultary Research Center of Biomaterials (CEIB), University of Liège, Chemistry Institute, Liège (Sart-Tilman), Belgium*
[g]*Instituto de Investigaciones Químicas (IIQ) CSIC-US, Centro de Investigaciones Científicas Isla de La Cartuja, Americo Vespucio 49, ES-41092 Sevilla, Spain*
[h]*Unidad de Enfermedades Infecciosas y Microbiología, Instituto de Investigación Biosanitaria ibs. Granada, Hospitales Universitarios de Granada/Universidad de Granada, Granada, Spain*

[1]Present address for J.M.: *Instituto de Higiene e Medicina Tropical (IHMT), Rua da Junqueira 100, 1349-008 Lisboa, Portugal.*

[2]Present address for J.J.V.-D.: *Department of Forest Products Technology, School of Chemical Technology, Aalto University, P.O. Box 16300, FI-00076 Aalto, Finland.*

[3]Present address for P.U.: *European Commission, Joint Research Centre, Institute for Health and Consumer Protection, IT-21027 Ispra (VA), Italy.*

This work was supported by grants BIO2011-25039, BIO2014-52872-R and CTQ2012-32605 from the *Ministerio de Economía y Competitividad*, Spain, which included FEDER funds, and 2014-SGR-938 from the *Generalitat de Catalunya*, Spain.

Patent application: Heparin-lipidic nanoparticle conjugates. Inventors: Fernàndez-Busquets, X., Marques, J., Moles, E. Institutions: IBEC, ISGlobal. Application number: EP13152187.4; priority country: Europe; priority date: January 22, 2013.

*Corresponding author at: Nanomalaria Unit, Centre Esther Koplowitz, 1st floor, ISGlobal, Rosselló 149-153, Barcelona ES08036, Spain.

Ph. +34 93 227 5400 (ext 4581).

E-mail address: xfernandez_busquets@ub.edu

Supplementary Materials

Full Methods

Materials

Except where otherwise indicated, reactions were performed at room temperature (20 °C), reagents were purchased from Sigma-Aldrich Corporation (St. Louis, MO, USA), and cultures of the *Plasmodium falciparum* 3D7 strain have been used. The lipids (all ≥99% purity according to thin layer chromatography analysis) 1,2-dioleoyl-*sn*-glycero-3-phosphocholine (DOPC), L-α-phosphatidylethanolamine (PE), 1,2-dipalmitoyl-*sn*-glycero-3-phosphoethanolamine-N-(4-(p-maleimidophenyl)butyramide (MPB-PE), 1,2-dioleoyl-*sn*-glycero-3-phosphoethanolamine-N(lissamine rhodamine B sulfonyl) (DOPE-Rho), and 1,2-dioleoyl-3-trimethylammonium-propane (DOTAP) were purchased from Avanti Polar Lipids Inc. (Alabaster, AL, USA).

Liposome preparation

Liposomes were prepared by the lipid film hydration method.[1] Lipids were dissolved in chloroform:methanol (2:1 v/v) in a round-bottom flask from where organic solvents were removed by rotary evaporation under reduced pressure at a temperature higher than the lipid melting point (i.e. 37 °C except for DOTAP-containing liposomes which were prepared at 40 °C) to yield a thin lipid film on the walls of the flask. Remaining solvent traces were eliminated by drying under N_2 flow for 30 min. The dry lipids were hydrated in phosphate-buffered saline (PBS) at 37 °C to obtain a concentration of 10 mM lipid and multilamellar liposomes were formed by 3 cycles of constant vortexing followed by bath sonication for 4 min each. Multilamellar liposomes were downsized to form uni- or oligolamellar vesicles by extrusion through 200-nm polycarbonate membranes (Poretics, Livermore, CA, USA) in an extruder device (LiposoFast, Avestin, Ottawa, Canada). Liposome size was determined by dynamic light scattering (DLS) using a Zetasizer NanoZS90 (Malvern Ltd, Malvern, UK).

Immunoliposome preparation

The mild reducing agent 2-mercaptoethylamine-HCl (MEA, Pierce Biotechnology) was used to generate half-antibodies following established protocols.[2] 90 µL of a 0.1 mg/mL PBS solution of the mouse monoclonal IgM BM1234 antibody (Acris Antibodies, Herford, Germany) targeted against pRBCs was added to 10 µL of 10× reaction buffer (PBS containing 5 mM EDTA and 50 mM MEA), and incubated for 90 min at 37 °C in a water bath. Unreacted MEA was separated from reduced half-antibodies by molecular exclusion chromatography (Zeba desalt spin columns, Pierce Biotechnology). Liposomes containing in their formulation the thiol-reacting lipid MPB-PE (DOPC:cholesterol:MPB-PE:DOPE-Rho, 72:20:1:7) were incubated with half-antibodies (0.01 µg/µL, 133 nM half-antibody assuming complete reduction by MEA) overnight at 4 °C. The resulting immunoliposomes were pelleted by ultracentrifugation (100,000 ×*g*, 90 min, 4 °C), and finally resuspended in 10 vol of PBS and kept at 4 °C for up to 2 weeks before use. DLS characterization of these liposomes (Figure S1) indicated that their mean size and polydispersity were similar to those previously described.[2]

Preparation of primaquine-containing liposomes functionalized with covalently bound heparin

The antimalarial drug primaquine (PQ) was encapsulated in DOTAP-containing liposomes (DOPC:cholesterol:PE:DOTAP, 46:20:30:4) by dissolving it at 1.2 mM in the PBS buffer used to hydrate the lipids, removing non-encapsulated drug by ultracentrifugation (150,000 ×*g*, 1 h, 4 °C). To crosslink the primary amine groups present in liposomal PE with the carboxyl groups of heparin (sodium salt from porcine intestinal mucosa, 13 kDa mean molecular mass) or its hexa- and octasaccharide fragments (Iduron, Cheshire, UK), the polymers were first dissolved at 1 mg/mL in MES activation buffer: 0.5 M NaCl, 0.1 M 2-(N-morpholino)ethane sulfonic acid, pH 5.0. Final concentrations of 2 mM N-(3-dimethylaminopropyl)-N'-ethylcarbodiimide hydrochloride (EDC, Fluka) and 5 mM N-

hydroxysuccinimide (NHS, Fluka) were added to the activated heparin solution. To obtain the desired heparin:liposome ratios, after 15 min the corresponding heparin solution and liposome suspension volumes in PBS buffer were mixed and incubated for 2 h with gentle stirring. To remove unbound heparin, liposomes were pelleted by ultracentrifugation (150,000 ×*g*, 1.5 h, 4 °C), and taken up in 10 pellet volumes of PBS immediately before addition to pRBC cultures with a further ca. 20-fold dilution (to obtain 3 µM final PQ concentration in the culture). For the quantification of encapsulated PQ, a lipid extraction of the liposomes was performed. Briefly, following ultracentrifugation the liposome pellet was treated with methanol:chloroform:0.1 M HCl (1.8:2:1) and after phase separation the PQ content in the upper water-methanol phase was determined by measuring A_{320} against a calibration curve of known PQ concentrations. DLS characterization (Figure S1) indicated that liposome mean size and polydispersity were similar to those previously described.[3] PQ release from these liposomes containing 80% of unsaturated lipids had also been characterized,[4] showing that ca. 70% of PQ remained encapsulated after 48 h in culture conditions. DOTAP-containing liposomes had been shown to lack non-specific toxicity up to 250 µM total lipid according to *in vitro* assays in human umbilical vein endothelial cell cultures.[3]

Heparin and chondroitin 4-sulfate (CSA) determination

Heparin and CSA concentrations were determined by the Alcian Blue method.[5] Shortly, 10 µL of GAG-containing solution was mixed with 10 µL of a solution containing 27 mM H_2SO_4, 0.375% Triton X-100, and 4 M guanidine-HCl, to which 100 µL of 1 mg/mL Alcian Blue 8GX solution in 0.25% Triton X-100, 18 mM H_2SO_4 were added. Samples were centrifuged and the pellet was resuspended in 500 µL of 8 M guanidine-HCl. Finally, after spinning down debris, A_{600} of the supernatant was recorded and carbohydrate content was determined from a standard linear regression of known heparin and CSA concentrations. Unspecific cytotoxicity and hemolysis assays were performed as described previously.[2]

Chitosan nanoparticle synthesis

Chitosan nanoparticles were prepared by a coacervation method described elsewhere.[6] Briefly, 0.5 g chitosan (low molecular weight, 75-85% deacetylated, Aldrich Ref. 448869) was dissolved in 50 mL of an aqueous solution of 2% v/v acetic acid containing 1% w/v Pluronic® F-68. About 12.5 mL of a 20% w/v sodium sulfate solution was added dropwise (2.5 mL/min) to the chitosan solution under mechanical stirring (1200 rpm) for 1 h to obtain a suspension of chitosan nanoparticles. The colloidal suspension was then subjected to a cleaning procedure that included repeated cycles of centrifugation (40 min, 14,000 ×*g*; Centrikon T-124 high-speed centrifuge, Kontron, Paris, France) and re-dispersion in water, until the conductivity of the supernatant was ≤10 μS/cm. Particle size was determined by photon correlation spectroscopy using a Malvern 4700 analyzer (Malvern Ltd). The measurement was made under a 60° scattering angle of the aqueous nanoparticle suspensions (0.1% w/v). The electrophoretic mobility measurements were performed in 0.1% w/v aqueous suspensions of nanoparticles in 1 mM KNO_3, pH 7.0, using a Malvern Zetasizer 2000 electrophoresis device (Malvern Ltd), under mechanical stirring (50 rpm) at 25 °C. The electrophoretic mobility was converted into zeta potential (ζ, mV) values as described by O'Brien and White.[7]

Determination of chitosan-heparin interaction

Isothermal titration calorimetry (ITC) measurements were performed with a VP-ITC microcalorimeter. The working cell was filled with chitosan or a chitosan nanoparticle suspension at a concentration of 0.1 mg/mL in PBS and the reference cell with the corresponding nanoparticle-free PBS solution. 10-μL aliquots of 1 mg/mL heparin solution in PBS were injected stepwise into the working cell at 200-s intervals, except for the first addition volume (2 μL), which was not used in the fit. As a reference blank experiment heparin solution was titrated in PBS in the absence of chitosan. The sample cell was constantly stirred at 300

rpm, and the measurements were performed at 25 °C. Data analyses were carried out with Origin software provided by MicroCal.

For fluorescence determinations, chitosan nanoparticles (5 mg/mL) and heparin labeled with fluorescein isothiocyanate (heparin-FITC, Life Technologies) were mixed 10:1 w/w and incubated for 90 min with gentle orbital mixing. After a centrifuge step (100,000 ×*g*, 1 h, 4 °C) to remove unbound heparin, the pellet was taken up in PBS, its fluorescence measured ($\lambda_{ex}/_{em}$: 488/525 nm), and the corresponding concentration determined against a standard linear regression of known FITC concentrations. The fluorescence of the supernatant was also measured to confirm that it contained the fraction of heparin not associated with the nanoparticles.

Fluorescent labeling of CSA

In an adaptation of existing protocols,[8] CSA (5 mg, *M*r ca. 20 kDa) was dissolved at 10 mg/mL in 0.1 M MES buffer (pH 5.0) containing 80 mM each of EDC and NHS, and incubated for 3 h. The mixture was precipitated by the addition of cooled ethanol, and CSA activated in its carboxyl groups was separated from the ethanol solution containing excess EDC and its by-products by centrifugation at ca. 4,300 ×*g* for 2 min. The CSA-NHS ester was dissolved in PBS (1.8 mL), to which a solution of 2.5 mg of 5-(((2-(carbohydrazino)methyl)-thio)acetyl)-aminofluorescein (Invitrogen) in DMSO (0.1 mL) was added, and the reaction was allowed to proceed for 12 h. Unreacted aminofluorescein was removed by size exclusion chromatography with a Sephadex G-25 gel column, using 9:1 water:methanol as eluent. Fractions containing labeled CSA were finally collected and freeze-dried. For analysis of the purity of fluorescent CSA, aliquots of 1 g/L samples were applied to a thin-layer chromatography plate and developed for 10 min in propanol:water (3:1 v/v). Plates were visualized by ultraviolet irradiation (365 nm) to detect the removal of unconjugated

aminofluorescein. Fluorescence intensity of the polysaccharide was measured with a microplate reader, and the fluorescein content of the sample was determined against a standard linear regression of known aminofluorescein concentrations, obtaining a final fluorescein:carboxyl molar ratio of 0.7%.

Plasmodium falciparum cell culture

The *P. falciparum* strains 3D7 and CS2 (obtained through the Malaria Research and Reference Reagent Resource Center, MR4, as part of the BEI Resources Repository, National Institute of Allergy and Infectious Diseases, National Institutes of Health; the CS2 strain, MRA-96, was deposited by SJ Rogerson) were grown *in vitro* in group B human RBCs using previously described conditions.[9] Briefly, parasites (thawed from glycerol stocks) were cultured at 37 °C in Petri dishes containing RBCs in Roswell Park Memorial Institute medium (RPMI-1640) supplemented with Albumax II (Invitrogen) and 2 mM L-glutamine, under a gas mixture of 92% N_2, 5% CO_2, and 3% O_2. Synchronized cultures were obtained by 5% sorbitol lysis, and the medium was changed every 2 days maintaining 3% hematocrit. For culture maintenance, parasitemias were kept below 5% late forms (trophozoites and schizonts) by dilution with washed RBCs prepared as described elsewhere.[2] For growth inhibition assays, parasitemia was adjusted to 1.5% with more than 90% of parasites at ring stage after sorbitol synchronization. 150 µL of this *Plasmodium* culture was plated in 96-well plates and incubated for 48 h in the conditions described above in the presence of test compounds that had been sterile-filtered (0.22 µm filters, Millipore). Parasitemia was determined by flow cytometry, after staining pRBC DNA with the nucleic acid dye Syto 11, added 10 min before analysis without any further washing step. Samples were analyzed using a BD FACSCalibur™ flow cytometer and parasitemia was expressed as the number of parasitized cells per 100 erythrocytes.

Fluorescence confocal microscopy

Living *P. falciparum* cultures with mature stages of the parasite were incubated in the presence of 7% DOPE-Rho-containing immunoliposomes (100 µM total lipid content in the dish) in PBS supplemented with 0.75% bovine serum albumin for 90 min at 37 °C with gentle stirring. After washing with PBS, blood smears were prepared and cells were fixed for 20 min with 1% v/v paraformaldehyde in PBS. Parasite nuclei were stained with 4'6-diamino-2-phenylindole (DAPI) and the RBC membrane was labeled with wheat germ agglutinin-Alexa Fluor 488 conjugate (Life Technologies). Slides were finally mounted with ProLong® Gold antifade reagent, and analyzed with a Leica TCS SP5 laser scanning confocal microscope. For fluorescein-labeled CSA-stained samples, mature stages of the *P. falciparum* CS2 parasite strain were incubated in the presence of 3.2 mg/mL fluorescent CSA for 90 min at 37 °C with gentle stirring. After washing 3× with PBS, parasite nuclei were stained with 10 µg/mL Hoechst 33342 (Molecular Probes, Ref. H-1399) for 30 min, and after 2 washing steps, the culture was finally diluted 10× in PBS and analyzed without fixation with a Leica TCS SP5 laser scanning confocal microscope. As a pRBC marker, hemozoin crystal reflection is shown in addition to DNA stain.

Cryo-transmission electron microscopy

1 mg/mL heparin-FITC was incubated with 2 mg/mL chitosan nanoparticles for 90 min with gentle stirring, and the resulting sample was centrifuged for 60 min at 4 °C and 100,000 ×*g* to remove unbound heparin. The nanoparticle-containing pellet was treated with a primary anti-FITC antibody (Rockland Immunochemicals Inc., Limerick, PA, USA), at a concentration of 5 µg/mL PBS for 1 h with gentle orbital stirring. After washing in PBS, the secondary anti-goat antibody conjugated to 6-nm colloidal gold (Jackson ImmunoResearch Laboratories Inc.) was added at a concentration of 50 µg/mL PBS and incubated for 90 min as above. Unbound

antibodies were removed by gel filtration chromatography through Sepharose CL-4B resin in a Micro Bio-Spin Column™ (Bio Rad). A thin aqueous film was formed by placing a 5 µL sample drop on a glow-discharged holey carbon grid and then blotting the grid against filter paper. The resulting thin films spanning the grid holes were vitrified by plunging the grid (kept at 100% humidity) into ethane, which was maintained at its melting point with liquid nitrogen, using a Vitrobot (FEI Company, Eindhoven, The Netherlands). The vitreous films were transferred to a Tecnai F20 transmission electron microscope (FEI Company) using a Gatan cryotransfer (Gatan, Pleasanton, CA, USA), and the samples were observed in a low dose mode. Images were acquired at 200 kV at a temperature between –170 and –175 °C, using low-dose imaging conditions not exceeding 20 e^{-}/$Å^{2}$, with a CCD Eagle camera (FEI Company).

In vitro coagulation test

Human blood from healthy volunteer donors was collected in Terumo Venosafe citrated tubes (Terumo Europe N. V., Belgium) and used within 2 h after blood collection. All tests were performed with the agreement of the local ethical committee from the Medicine Faculty at the University of Liège. Whole blood and GAG-containing samples were mixed and incubated for 15 min at 37 °C, centrifuged at 2,000 ×*g* for 5 min, and the supernatants were collected, recalcified to reverse the effect of citrate anticoagulant, and supplied with the specific activators of coagulation (thromboplastin). Prothrombin time, to evaluate the extrinsic pathway, was measured directly with a Dade Behring Coagulation Timer analyzer (Siemens Healthcare Diagnostics NV/SA, Belgium) using commercial reagents (Thromborel® S, Dade Behring/Siemens). Kaolin reagent was used as a positive control and PBS as a negative control. Clotting time was measured for each sample, and coagulation capacity was expressed as a percentage, taking the value of standard human plasma (Dade Behring/Siemens) as 100%. Measurements were done in duplicate with differences between both replicas <1%. Coagulation equipment was programmed to perform at least 2 measurements on the same

samples, and if the difference between them was below 5% the analysis was not repeated; otherwise, it was repeated once more.

Force spectroscopy

Binding forces between CSA and pRBCs infected with the *P. falciparum* CS2 strain were measured with an MFP-3D atomic force microscope (Asylum Research, Santa Barbara, CA, USA). CSA molecules were immobilized on the tip of NP-S cantilevers (Veeco Instruments Inc.; spring constants in the range 0.05-0.08 N m^{-1} were obtained by thermal method) that were previously silanized in vapour phase with 3-aminopropyl triethoxysilane (Fluka, Buchs, Switzerland). The immobilization via covalent bonds between carboxyl groups of the CSA molecules and amine groups of the silanized cantilevers took place by immersing the cantilevers in a solution of 100 µg/mL CSA containing 2.5 mM EDC and 10 mM NHS for about 1 h, followed by rinsing with PBS. In parallel to CSA immobilization, 50 µL of mature stage pRBCs purified by magnetic cell sorting (MACS columns, Miltenyi Biotec) and suspended in RPMI medium were deposited on poly-L-lysine-coated glass slides (StarFrost, Waldemar Knittel Glasbearbeitungs GmbH, Braunschweig, Germany), which were prepared in advance by coating them with 0.01% poly-L-lysine solution for 30 min followed by rinsing with double deionised water (Milli-Q system, Millipore) and drying by evaporation. After about 1 h of adsorption, weakly and non-attached pRBCs were removed by gently rinsing the glass slides several times with 100 µL PBS. Force curves were acquired in PBS by approaching the cantilever tip with immobilized CSA to the pRBCs adsorbed on the glass slide and retracting it after contact. The approaching velocity was kept constant at 3 µm s^{-1}, whereas for dynamic force spectroscopy the retraction speed was varied between 3 and 14.5 µm s^{-1}. The corresponding loading rates were calculated by multiplying the retraction velocities by the effective spring constant of the system,[10] which was ca. 10% of the spring constants of the cantilevers. Maximum applied forces were below 0.5 nN to prevent cell lysis.[11] In a typical

experiment, between 500 and 2300 force curves were collected in 25-115 different spots on the same or different cells. Adhesion between CSA and pRBC was evaluated from the unbinding events in the retraction force curves. When several unbinding events were observed, only the last one was considered for analysis. Force histograms were obtained by plotting the binding probability for different binding forces. The binding probability for a certain binding force was calculated as the percentage of force curves (relative to the total number of force curves) showing that particular binding force. Force histograms were fitted to Gaussian or 2-peak Gaussian functions to obtain the average binding forces. Control experiments with non-infected RBCs were also performed.

P. falciparum gametocyte culture and targeting assay

Gametocytes of the *P. falciparum* NF54 strain (obtained through the MR4 as part of the BEI Resources Repository) were obtained from continuously maintained cultures of asexual blood stage parasites, setting up flasks at 1% parasitaemia and 3% hematocrit. Gametocytes began to form in significant numbers in blood culture following daily medium change. All operations were performed on a warming plate set to 37/38 °C in order to minimize heat loss to cultures during the time they were out of the incubator. Smears and an exflagellation test were performed on culture days 4, 7, 10, and 14. For the exflagellation test, a few µL of the culture were spun down and, after discarding the supernatant, the pellet was taken up in 5 µL of ookinete medium (see below) and added to a disposable counting slide; after 20 min incubation at room temperature the slide was observed under the microscope with the 40× objective. For targeting assays, 10 mL of a *P. falciparum* stage V gametocyte culture was centrifuged (37 °C, 5 min, 500 ×*g*), and the culture pellet was taken up in 3 mL of RPMI medium and added (1:1 v/v) to a heparin solution in the same buffer. Samples were incubated in the presence of 0.25 mg/mL heparin-FITC for 90 min at 37 °C, and the cultures were subsequently spun down and

washed 3 times with RPMI. Smears were fixed with 4% paraformaldehyde, stained with DAPI, and examined by fluorescence confocal microscopy.

Plasmodium berghei ookinete culture and targeting assay

Ookinete culture medium consisted of 16.4 g/L RPMI supplemented with 2% w/v $NaHCO_3$, 0.05% w/v hypoxanthine, 100 µM xanthurenic acid, 50 U/mL penicillin, 50 µg/mL streptomycin (Invitrogen), 25 mM HEPES, pH 7.4. Complete medium was prepared just before use by supplementing with heat-inactivated fetal bovine serum (FBS, Invitrogen) to a final concentration of 20%. Six days prior to performing the targeting assay, a mouse was treated intraperitoneally with 10 µg/mL phenylhydrazine (PHZ) to induce reticulocytosis. Three days after PHZ treatment the mouse was inoculated by intraperitoneal injection of 200 µL of blood containing ca. 5×10^7 *P. berghei* mCherry (a kind gift from Dr. D. Vlachou) pRBCs extracted by cardiac puncture from a donor mouse that had been infected intraperitoneally 3 days before with 200 µL of a cryopreserved *P. berghei* suspension just thawed. Three days later, 1 mL of infected blood was collected by cardiac puncture onto 30 mL ookinete medium, and incubated for 24 h at 19-21 °C with 70-80% relative humidity. For ookinete targeting assays, 100 µL of 0.25 mg/mL heparin-FITC were added to 100 µL of culture and incubated in the dark for 90 min under orbital stirring (300 rpm). The samples were centrifuged for 1.5 min at 800 ×*g* and washed 3× with PBS. Fixed cell slides were prepared by adding 0.5 µL FBS to 0.5 µL pellet and by fixing the smear with 4% paraformaldehyde for 15 min. After performing 3 washing steps with PBS, slides were mounted with Vectashield® DAPI-containing media (Vector Laboratories, UK). All work involving laboratory animals was performed with humane care in accordance with EU regulations (EU Directive 86/609/EEC) and with the terms of the United Kingdom Animals (Scientific Procedures) Act (PPL 70/8788), and was approved by the Imperial College Ethical Review Committee.

P. berghei oocyst culture and targeting assay

Six days prior to feeding female *Anopheles stephensi* mosquitoes, a mouse was treated intraperitoneally with PHZ as described above, and three days after treatment the mouse was infected by intraperitoneal injection with *P. berghei* mCherry pRBCs (a kind gift from Dr. D. Vlachou). The selected female mosquitoes were not fed with sugar in the 24 h preceding the blood feed but were kept hydrated with water. On the day of the feed, the parasitemia and gametocytemia of the mouse were recorded, and an exflagellation test was also performed. Subsequently the mouse was anesthetized and placed on the netting of the mosquito cage. The feed was maintained for 30 min at 19-21 °C in the dark. After feeding, as there was a pool of sticky blood on the bottom of the cage, the pot was laid on its side overnight at 19-21 °C with 70-80% humidity. On day 1 post-feeding, the mosquitoes were anesthetized with CO_2 and kept on ice while the unfed ones were removed. Mosquitoes were then fed with 5% glucose/0.05% 4-aminobenzoic acid every 2 days and maintained for 21 days at 19-21 °C and 70-80% humidity.

For oocyst targeting assays mosquitoes were dissected for midguts on day 10 post-feeding. After anesthetizing the mosquitoes with CO_2 and keeping them on ice, they were added to ~300 µL RPMI medium and the midguts were removed under a dissecting microscope and carefully collected onto a 24-well plate containing 500 µL RPMI (5 midguts/well). The guts were transferred to a well containing heparin-FITC dissolved in RPMI at 0.25 mg/mL, and incubated for 90 min at room temperature with gentle stirring. Samples were then washed twice with PBS, and fixed with 4% paraformaldehyde for 15 min. After fixation, two washing steps with PBS were performed, and the slides were finally prepared by transferring the midguts to a slide containing a few µL of Vectashield® antifade mounting medium containing DAPI.

P. berghei sporozoite culture and targeting assay

For sporozoite targeting assays, after performing direct *A. stephensi* feeding on malaria-infected mice as described above, mosquitoes were dissected for salivary glands on day 21 post-feeding. Glands were sliced from the head and transferred to an eppendorf tube containing 50 µL of RPMI medium. After centrifugation for 10 min at 2350 ×*g*, the pellet was taken up in 100 µL of 0.2 mg heparin-FITC/mL RPMI and incubated for 90 min at 37 °C with gentle stirring. Samples were finally centrifuged for 5 min at 2350 ×*g* and washed 3 times with RPMI. When the last wash was completed, the pellet was taken up in Hoechst 33342 solution (0.05 µg/mL) and a 5-min centrifugation step at 2350 ×*g* was performed. The pellet was finally taken up in RPMI and the slides containing live cells were prepared for confocal microscopy analyses.

Statistical analysis

Data are presented as the mean ± standard deviation of at least three independent experiments, and the corresponding standard deviations in histograms are represented by error bars. The parametric Student's t-test was used to compare two independent groups when data followed a Gaussian distribution, and differences were considered significant when $p \leq 0.05$. Percentages of viability were obtained using non-treated cells as control of survival and IC50 values were calculated by nonlinear regression with an inhibitory dose-response model using GraphPad Prism5 software (95% confidence interval). Concentrations were transformed using natural log for linear regression, and regression models were adjusted for the assayed replicates.

Supplementary Figures

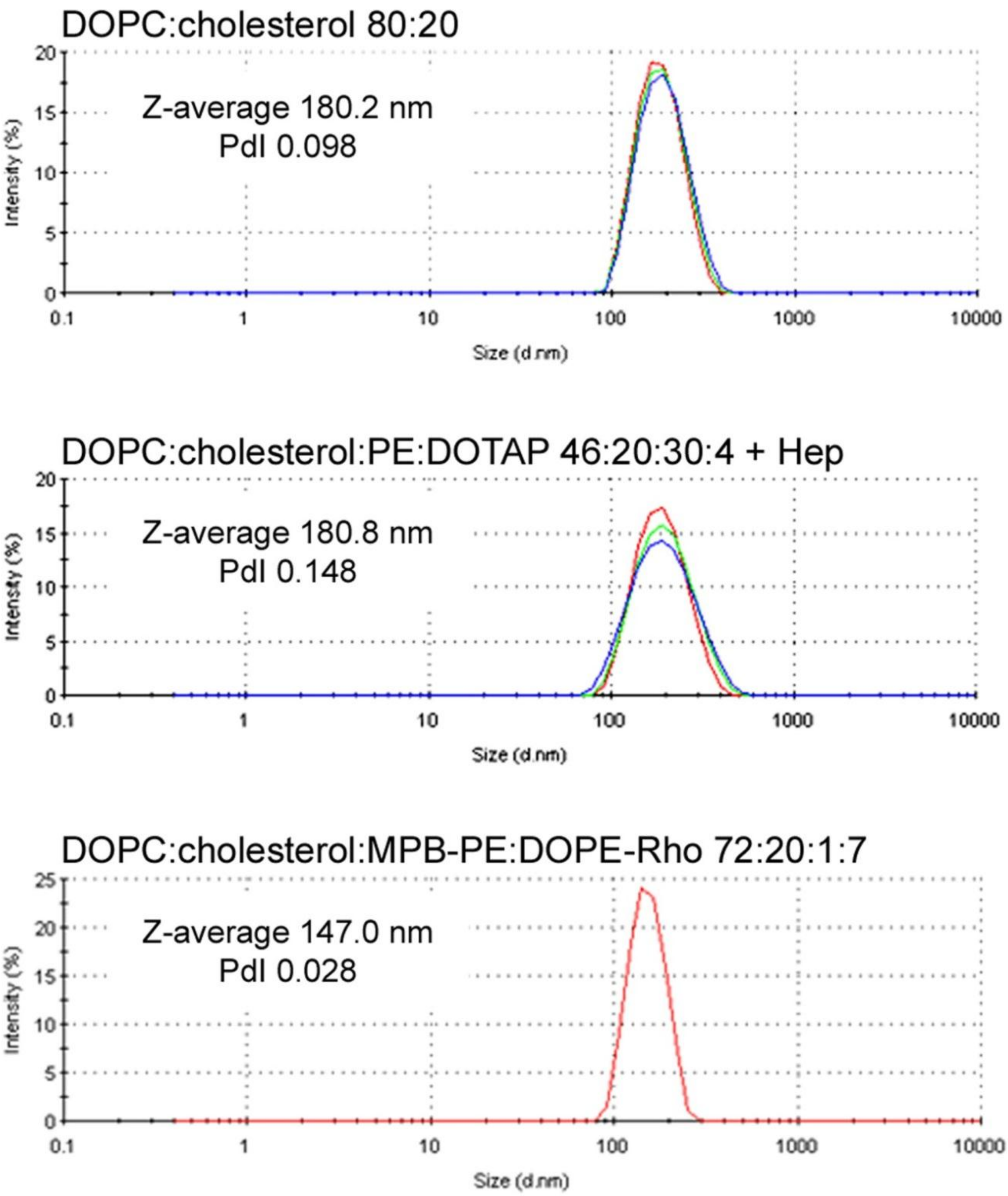

Figure S1. DLS analysis of control DOPC:cholesterol 80:20 liposomes and of the two types of liposomes used in this work: DOTAP-containing liposomes for heparin targeting and MPB-PE-containing immunoliposomes labeled with rhodamine. PdI: polydispersity index.

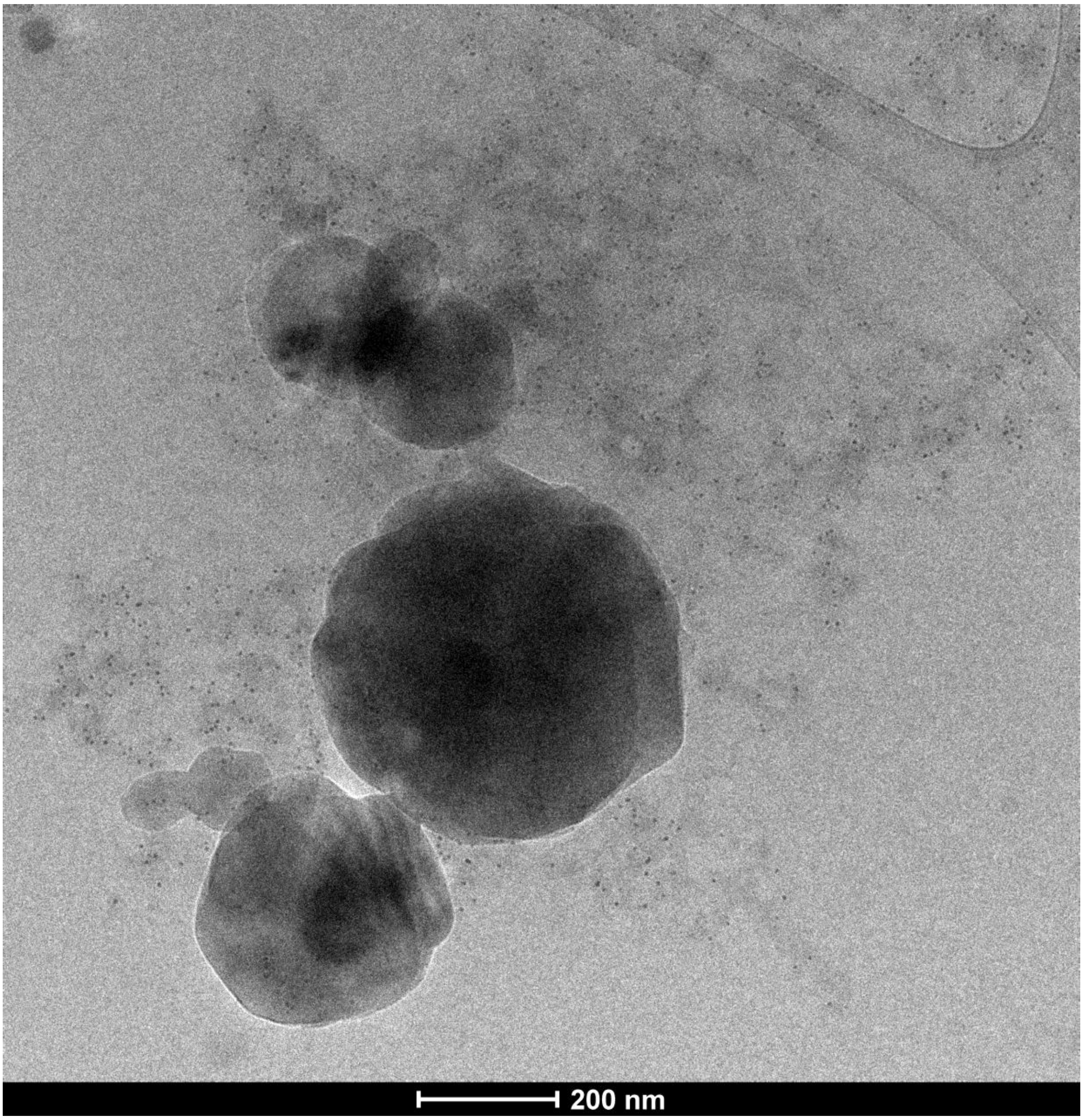

Figure S2. Cryo-transmission electron microscopy image of heparin-FITC bound to chitosan nanoparticles.

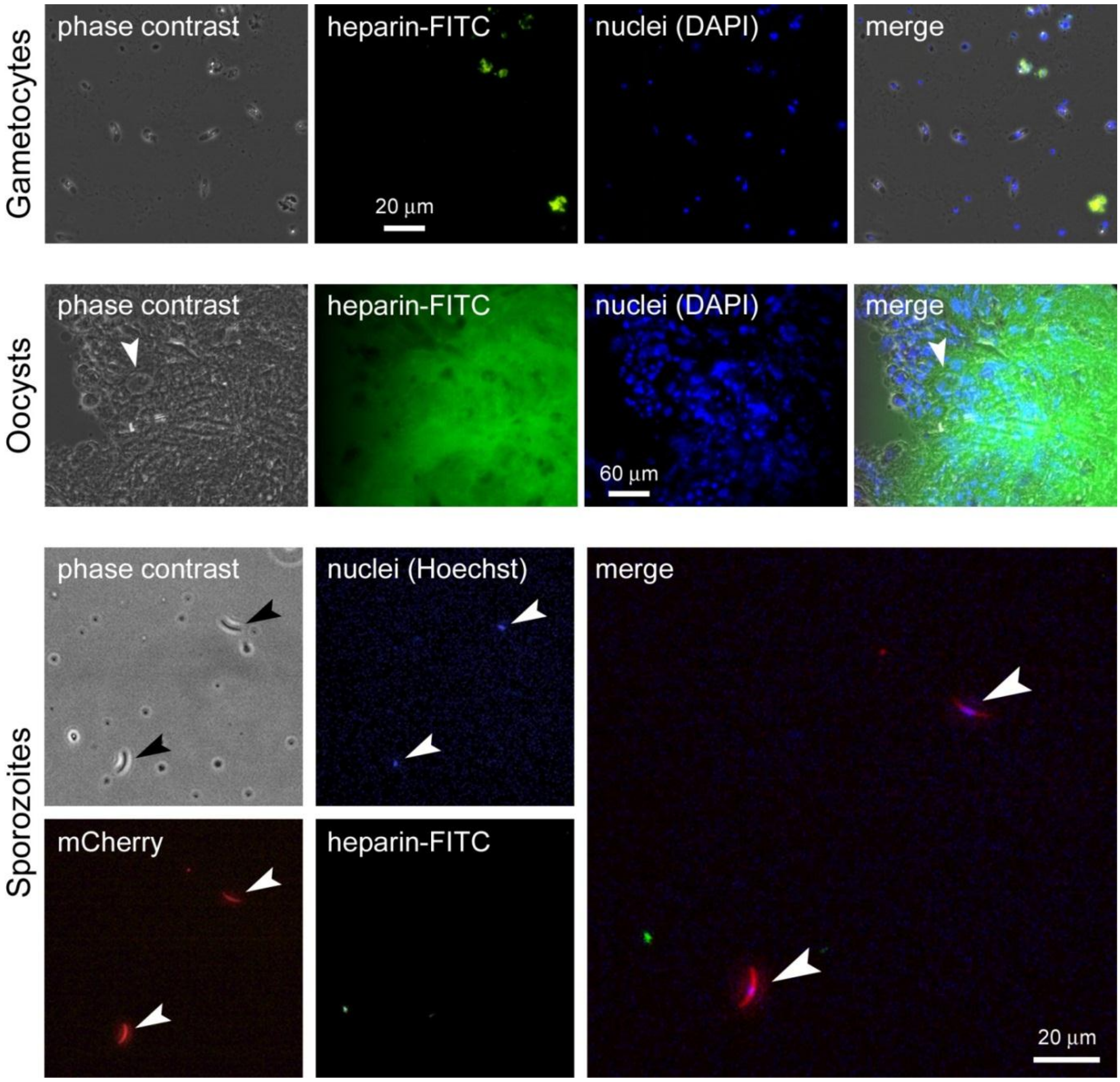

Figure S3. Fluorescence confocal microscopy analysis of the binding of heparin-FITC to living *P. falciparum* stage V gametocytes and *P. berghei* oocysts and sporozoites. Arrowheads indicate one oocyst and two sporozoites.

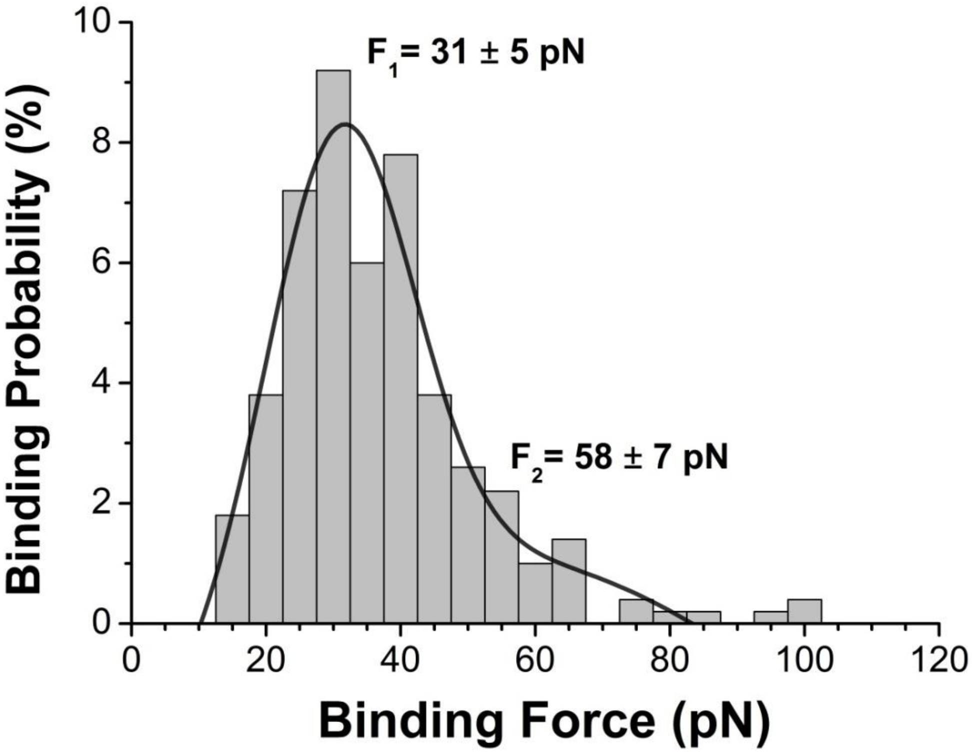

Figure S4. Representative force histograms for the binding of CSA to pRBCs in the presence of 500 µg/mL CSA in solution, at a loading rate of 21.5 nN s^{-1}. The solid line corresponds to a 2-peak Gaussian fit.

Supplementary Video

Living *P. falciparum* cultures with mature stages of the parasite were incubated in glass bottom dishes (MatTek Corporation) in the presence of immunoliposomes containing in their formulation 1% DOPE-Rho (red fluorescence; 400 µM total lipid content in the dish) targeted to pRBCs (identified by Hoechst 33342 blue fluorescence). Phase contrast imaging is used to show the erythrocytes during the 12 h of duration of the assay.

References

1. MacDonald RC, MacDonald RI, Menco BP, Takeshita K, Subbarao NK, Hu LR. Small-volume extrusion apparatus for preparation of large, unilamellar vesicles. *Biochim Biophys Acta* 1991; **1061(2)**:297-303.
2. Urbán P, Estelrich J, Cortés A, Fernàndez-Busquets X. A nanovector with complete discrimination for targeted delivery to *Plasmodium falciparum*-infected versus non-infected red blood cells *in vitro*. *J Control Release* 2011; **151(2)**:202-211.
3. Marques J, Moles E, Urbán P, Prohens R, Busquets MA, Sevrin C*, et al.* Application of heparin as a dual agent with antimalarial and liposome targeting activities towards *Plasmodium*-infected red blood cells. *Nanomedicine: NBM* 2014; **10**:1719-1728.
4. Moles E, Urbán P, Jiménez-Díaz MB, Viera-Morilla S, Angulo-Barturen I, Busquets MA, *et al.* Immunoliposome-mediated drug delivery to *Plasmodium*-infected and non-infected red blood cells as a dual therapeutic/prophylactic antimalarial strategy. *J Control Release* 2015; **210**:217-229.
5. Frazier SB, Roodhouse KA, Hourcade DE, Zhang L. The quantification of glycosaminoglycans: a comparison of HPLC, carbazole, and Alcian Blue methods. *Open Glycosci* 2008; **1**:31-39.
6. Arias JL, López-Viota M, Gallardo V, Ruiz MA. Chitosan nanoparticles as a new delivery system for the chemotherapy agent tegafur. *Drug Dev Ind Pharm* 2010; **36(6)**:744-750.
7. O'Brien RW, White LR. Electrophoretic mobility of a spherical colloidal particle. *J Chem Soc, Faraday Trans 2* 1978; **74(0)**:1607-1626.
8. Han ZR, Wang YF, Liu X, Wu JD, Cao H, Zhao X, *et al.* Fluorescent labeling of several glycosaminoglycans and their interaction with anti-chondroitin sulfate antibody. *Chin J Anal Chem* 2011; **39(9)**:1352-1357.

9. Cranmer SL, Magowan C, Liang J, Coppel RL, Cooke BM. An alternative to serum for cultivation of *Plasmodium falciparum in vitro*. *Trans R Soc Trop Med Hyg* 1997; **91(3)**:363-365.

10. Fuhrmann A, Anselmetti D, Ros R, Getfert S, Reimann P. Refined procedure of evaluating experimental single-molecule force spectroscopy data. *Phys Rev E* 2008; **77(3)**:031912.

11. Hategan A, Law R, Kahn S, Discher DE. Adhesively-tensed cell membranes: lysis kinetics and atomic force microscopy probing. *Biophys J* 2003; **85(4)**:2746-2759.
